# Comprehensive Oxidation Mechanism of *n*‑Butylamine and 2‑Butylamine by H and OH Radicals: Insights into Reactivity

**DOI:** 10.1021/acs.jpca.5c02556

**Published:** 2025-06-02

**Authors:** Joel Leitão Nascimento, Tiago Vinicius Alves, Yanlei Shang

**Affiliations:** † Departamento de Físico-Química, 28111 Instituto de Química, Universidade Federal da Bahia, Rua Barão de Jeremoabo, 147, 40170-115- Salvador, Bahia, Brazil; ‡ Energy Research Institute, Qilu University of Technology (Shandong Academy of Sciences), Jinan, Shandong 250014, P. R. China; § School of Materials Science and Engineering, 12689 Southwest Jiaotong University, Chengdu, Sichuan 610031, P. R. China

## Abstract

This study presents the accurate thermal rate constants for a series of hydrogen abstraction reactions involving 1- and 2-butylamine and key radicals H and OH. The potential energy surface resulting from these reactions was examined by using the M08-HX/ma-TZVP level of theory. The rate coefficients were calculated within the multistructural canonical variational theory with small-curvature tunneling correction (MS-CVT/SCT). Multistructural effects and the torsional anharmonicity corrections were evaluated through the rovibrational partition function calculated with the multistructural method based on a coupled torsional potential (MS-T). Our results demonstrated an influence of the position of the amino functional group on the kinetics. The gradual decrease in barrier heights was observed with increasing distance between the amino functional group and the reaction site. The calculated branching ratios demonstrated that the H-abstraction by the H radicals at the α-site is favored. In reactions involving OH radicals, the channel at the N-site shows a greater proportion due to its increased multistructural torsional anharmonicity and a reduced variational effect of other sites.

## Graphical abstract

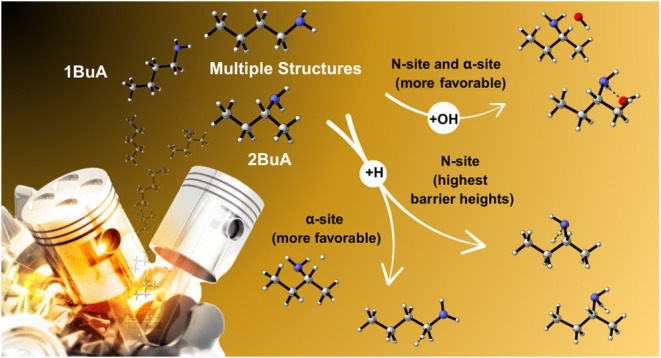

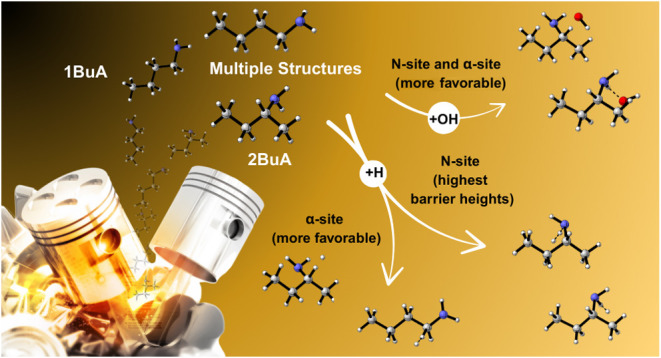

## Introduction

The increasing demand for fossil fuels has resulted in increased atmospheric pollution due to the emissions produced by their combustion, which include volatile organic compounds (VOCs) and greenhouse gases.^1^ To address these environmental issues, various global initiatives are promoting sustainable alternatives, such as e-fuels,^2,3^ second-generation biofuels,^4,5^ and ethanol,^6^ among others. These renewable fuels can help reduce emissions and can also be blended with petroleum derivatives to enhance the research octane number (RON).^7^

A promising class of biofuels includes nitrogen-based compounds, which are recognized for their lower soot formation, potential to reduce CO_2_ emissions, and natural presence in the molecular structure of biomass.^8–10^ Ammonia, for example, is considered a strong candidate for clean fuel generation and is often studied as an additive for high-reactivity fuels.^11–14^ Other substances, such as ethylhexyl nitrate, have also been investigated as diesel additives.^15^ Experimental and theoretical investigations have been conducted to better understand the combustion behavior of nitrogenous compounds, particularly focusing on the C1–C3 aliphatic amines. These include methylamine (MA),^16,17^ ethylamine (EA),^16,18,19^ dimethylamine,^19–21^ *n*-propylamine (NPA),^22^ and *iso*-propylamine.^23^ The combustion mechanism of nitrogen-containing compounds is quite complex, and to fully understand their reactivity, pollutant emissions, and viability as biofuels, it is essential to reassess these compounds by using larger molecules.

Recently, Montgomery et al.^8^ conducted a detailed analysis of how nitrogen-containing fuels influence soot formation, comparing them to oxygenated compounds and hydrocarbons. Their analysis revealed that *n*-butylamine, when compared to *n*-butanol, resulted in a reduction of up to 7.0% in yield sooting indices (YSIs), indicating a lower impact on soot formation. The authors found that in primary amines the position of the amine group on the carbon atom affects the tendency for soot formation among constitutional isomers. For instance, 2-butylamine, *tert*-butylamine, and *iso*-butylamine show a greater propensity to emit soot compared to *n*-butylamine. This relationship between soot formation and the chemical structure of amines highlights the complexity of these compounds and points to an area that is still underexplored in combustion chemistry.

Nitrogen-containing compounds show promise as potential biofuels; however, their combustion is likely to generate nitrogenous pollutants, such as hydrogen cyanide and nitrogen oxides (NOx), which pose significant threats to air quality.^24^ Despite this concern, as reported by Montgomery et al.,^8^ compounds like *n*-butylamine may still be viable options for biofuels, even in challenging conditions. This highlights the importance of thoroughly understanding the mechanisms involved in the combustion of *n*-butylamine and its isomers. Specifically, it is crucial to focus on their reactivity with radicals, such as hydrogen (H) and hydroxyl (OH), which play a key role in the initial stages of fuel consumption.

Despite their relevance, accurate kinetic parameters for the combustion mechanisms of *n*-butylamine and its isomers are scarce in the literature. To the best of our knowledge, only a few studies have reported the thermal rate constants for the hydrogen abstraction reactions of this amine and its isomers. Yan et al.^25^ performed a detailed investigation of the hydrogen abstraction reactions of *n*-butylamine initiated by H/CH_3_/NO_2_ and the subsequent β-scission from intermediates formed during these reactions. The authors employed a dual-level electronic structure approach, performing geometry optimizations and harmonic vibrational frequency calculations using an M06–2*X*/6–311++G­(d,p). Energetic refinements were performed with the high-level CCSD­(T)/CBS_D‑T_ approach. Additionally, rate constants were estimated using transition state theory (TST) and Rice–Ramsperger–Kassel–Marcus (RRKM) theory in the temperature range of 300–2000 K. Their results indicated that the H-abstraction by H atoms is more favorable compared to the CH_3_ radical. Previous experimental/theoretical^26,27^ investigations studied the oxidation of *tert*-butylamine by the hydroxyl radicals, focusing on the formation of nitrosamines and nitramines.

In this context, this work systematically aims to understand the influence of hydrogen abstraction at different sites of *n*-butylamine (hereafter referred to as 1-butylamine, 1BuA) and 2-butylamine (2BuA) by H and OH radicals. For 1BuA, the H-abstraction can occur at the nitrogen and the carbons α, β, γ, and δ (Figure 1a), leading to the following five reaction channels

$$\begin{array}{l} R^{1\mathrm{BuA}}x-1:1\mathrm{BuA}+H/\mathrm{OH}\to P_{N}+H_{2}/H_{2}O\\ R^{1\mathrm{BuA}}x-2:1\mathrm{BuA}+H/\mathrm{OH}\to P_{C_{\alpha }}+H_{2}/H_{2}O\\ R^{1\mathrm{BuA}}x-3:1\mathrm{BuA}+H/\mathrm{OH}\to P_{C_{\beta }}+H_{2}/H_{2}O\\ R^{1\mathrm{BuA}}x-4:1\mathrm{BuA}+H/\mathrm{OH}\to P_{C_{\gamma }}+H_{2}/H_{2}O\\ R^{1\mathrm{BuA}}x-5:1\mathrm{BuA}+H/\mathrm{OH}\to P_{C_{\delta }}+H_{2}/H_{2}O \end{array}$$

where *x* = 1 if the reaction occurs with H, and *x* = 2 if the abstraction proceeds with the OH radical.

**Figure 1:**
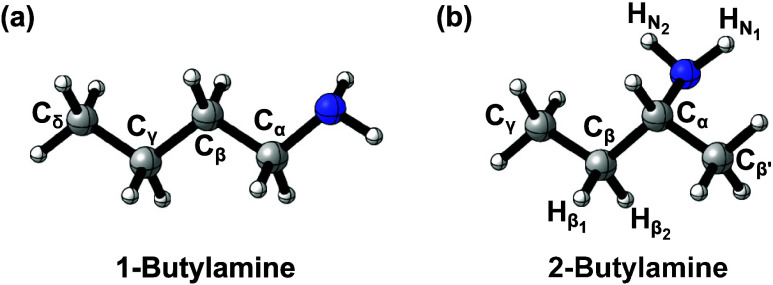
Three-dimensional representation of (a) 1-butylamine and (b) 2-butylamine with the abstraction sites labeled, where *x* equals 1 and 2 corresponding to H and OH radicals, respectively.

In the case of 2-butylamine, seven distinct reactions occur due to the system’s chirality, resulting in two different abstraction pathways. This behavior was also observed for 2-butanol, as reported by Ferro-Costas et al.^28^ The additional labels 1 and 2 stand for the hydrogen abstraction at the nitrogen (H_N1_ and H_N2_) and β-carbon (C_β1_ and C_β2_) sites (Figure 1b), respectively

$$\begin{array}{l} R^{2\mathrm{BuA}}x-1-1:2\mathrm{BuA}+H/\mathrm{OH}\to P_{N,1}+H2/H_{2}O\\ R^{2\mathrm{BuA}}x-1-2:2\mathrm{BuA}+H/\mathrm{OH}\to P_{N,2}+H_{2}/H_{2}O\\ R^{2\mathrm{BuA}}x-2:2\mathrm{BuA}+H/\mathrm{OH}\to P_{C_{\alpha }}+H_{2}/H_{2}O\\ R^{2\mathrm{BuA}}x-3-1:2\mathrm{BuA}+H/\mathrm{OH}\to P_{C_{\beta },1}+H2/H_{2}O\\ R^{2\mathrm{BuA}}x-3-2:2\mathrm{BuA}+H/\mathrm{OH}\to P_{C_{\beta },2}+H_{2}/H_{2}O\\ R^{2\mathrm{BuA}}x-4:2\mathrm{BuA}+H/\mathrm{OH}\to P_{C_{\gamma }}+H_{2}/H_{2}O\\ R^{2\mathrm{BuA}}x-5:2\mathrm{BuA}+H/\mathrm{OH}\to P_{C_{\beta }^{\prime }}+H_{2}/H_{2}O \end{array}$$

To obtain accurate results for the kinetic parameters, we investigated the potential surface generated by these reactions using the hybrid meta-GGA density functional M08-HX^29^ with the ma-TZVP^30^ basis set. The conformational flexibility and torsional anharmonicity were adequately considered through estimating the rovibrational participation function, employing the multistructural torsional anharmonicity method, MS-T.^31^ Thermal rate constants were determined with the help of the multistructural variational transition state theory^32–34^ with small-curvature tunneling. The overall thermal rate constants were calculated using the quasi-steady-state approximation, considering the effects of the reaction complex (RC) through the pre-equilibrium model (PEM). The product branching ratios were also estimated.

## Computational Details

### Electronic Structure Calculations

Given the significant conformational flexibility of 1-butylamine (1BuA) and 2-butylamine (2BuA), it is essential to conduct an exhaustive conformational search to identify the ground-state structures of reactants and transition states (TSs) for the H-abstraction reactions initiated by H and OH radicals. Conformation searches were performed using the ConfGen.exe code in the MSTor program.^35,36^ By calling the Gaussian 16 C01 program,^37^ the M08-HX/ma-TZVP^29,30^ method was selected for geometry optimizations and harmonic frequency calculations. The dihedral angles of all effective torsions in the molecular structures of reactants and TSs were rotated simultaneously by 0, 120, and −120° to generate the initial possible structures, as displayed in Figure S1. Due to the symmetry of the methyl (−CH_3_) groups, their rotations were not considered; however, their coupling effects with other torsions must be taken into account when selecting the distinguishable conformers.

In our previous work on H-abstraction reactions from a series of amines,^16,38–40^ the M08-HX/ma-TZVP method was examined as a good choice with a mean-unsigned deviation of <1.0 kcal mol^–1^ in comparison with the gold-standard CCSD­(T)/CBS method. Nevertheless, the performance of this density functional theory (DFT) method in describing the larger reaction systems requires further validation and quantification. Considering the size of the current reaction systems, we selected a relatively cost-effective yet widely validated method, DLPNO–CCSD­(T),^41^ as the high-level method. Combined with the cc-pV*X*Z basis sets (*X* = *T* and *Q*),^42^ the DLPNO–CCSD­(T) energies were extrapolated to the complete basis set (CBS) limit, DLPNO–CCSD­(T)/CBS­(T-Q), using eqs 1 and 2

1 $$E_{\mathrm{HF}}^{\mathrm{CBS}}=\frac{E_{\mathrm{HF}}^{\left(X\right)}e^{-\alpha \sqrt{Y}}-E_{\mathrm{HF}}^{\left(Y\right)}e^{-\alpha \sqrt{X}}}{e^{-\alpha \sqrt{Y}}-e^{-\alpha \sqrt{X}}}$$

2 $$E_{\mathrm{CC}}^{\mathrm{CBS}}=\frac{X^{\beta }E_{\mathrm{corr}}^{\left(X\right)}-Y^{\beta }E_{\mathrm{corr}}^{\left(Y\right)}}{X^{\beta }-Y^{\beta }}$$

where the α and β coefficients are 5.460 and 3.050, respectively, as recommended by Neese and Valeev.^43^ All of the DLPNO–CCSD­(T) calculations were carried out with the ORCA 5.0.0 program.^44^

### Rate Constant Calculations

The thermal rate constants of the H-abstraction reactions from 1BuA and 2BuA initiated by H and OH radicals can be directly calculated within the framework of multistructural canonical variational theory with small-curvature tunneling correction (MS-CVT/SCT)^32–34^ as follows

3 $$k^{\mathrm{MS}-\mathrm{CVT}/\mathrm{SCT}}=F^{\mathrm{MS}-T}\Gamma^{\mathrm{CVT}}\kappa^{\mathrm{SCT}}k^{\mathrm{TST}}$$

where *F* ^MS‑T^ is the multistructural torsional (MS-T) factor of the reaction; Γ^CVT^ and κ^SCT^ are the coefficients that account for the recrossing and tunneling transmission effects, respectively; and *k* ^TST^ is the rate constant obtained from conventional transition state theory (TST).^45^ These coefficients are temperature-dependent and require the construction of minimum energy paths (MEPs) based on the ground-state TSs. For this purpose, we constructed the MEPs using the Page–McIver algorithm^46^ with a step size of 0.005 Bohr, and the Hessian was estimated for every 10 steps. The frequencies were scaled by a factor of 0.976.^47^ For the H-abstraction reactions initiated by H radicals, each MEP was extended until κ^SCT^ converged with an error of <1%. The MEPs and *k* ^TST^ values were calculated at the M08-HX/ma-TZVP level of theory. All kinetic calculations were carried out using the Pilgrim code.^48,49^

The *F* ^MS‑T^ factor considers the contributions of multiple structures and their respective torsional anharmonicity in the partition functions of both reactants and TSs, i.e.

4 $$F^{\mathrm{MS}-T}=\frac{F^{\mathrm{MS}-T,‡}}{F^{\mathrm{MS}-T,R}}=\frac{Q_{\mathrm{rovib}}^{\mathrm{SS}-\mathrm{QH},R}}{Q_{\mathrm{rovib}}^{\mathrm{SS}-\mathrm{QH},‡}}\frac{Q_{\mathrm{rovib}}^{\mathrm{MS}-T,‡}}{Q_{\mathrm{rovib}}^{\mathrm{MS}-T,R}}$$

where *Q*_rovib_^SS‑QH^, *Y* (*Y* = *R* or ‡), is the rotational vibrational partition function of the single structure with the lowest energy determined by the classical rigid-rotor and quantum quasi-harmonic oscillator (QH) approximations; and *Q*_rovib_^MS‑T,Y^ is the multistructural rotational vibrational partition function based on a coupled torsional potential estimated by the MSTor program.^31^

For the H-abstraction reactions initiated by H radicals, their rate constants can be safely determined using eq 3. For the H-abstraction reactions initiated by OH radicals, a two-step process should be considered during rate constant calculations. The first step involves the formation of a prereactive complex (RC) due to strong intermolecular interactions, such as van der Waals forces or hydrogen bonding. The second step is a thermal activation process, in which the RC crosses TS to yield the final products. The total reaction process can be described as follows

$$1\mathrm{BuA}/2\mathrm{BuA}+\mathrm{OH}\overset{k_{1}}{\underset{k_{-1}}{⇌}}\mathrm{RC}\left[1\mathrm{BuA}/2\mathrm{BuA}\cdot \cdot \cdot \mathrm{OH}\right]\overset{k_{2}}{\to }\mathrm{products}$$

where *k*_1_ and *k*_–1_ represent the forward and reverse rate constants for RC formation, respectively, and *k*_2_ is the rate constant for the unimolecular decomposition of RC into products via a TS. By assuming the equilibrium between RC and reactants with *k*_–1_ being much larger than *k*_2_, the effective rate constant for this two-step process can be calculated using the pre-equilibrium model (PEM)

5 $$k_{\mathrm{PEM}}=K_{\mathrm{eq}}k_{2}$$

where *K*_eq_ is the equilibrium constant for RC formation from reactants and equals *k*_1_/*k*_–1_, and *k*_2_ can be determined using eq 3.

## Results and Discussion

### DFT Method Validation and MS-T Anharmonicity

Based on the optimized ground-state structures by the M08-HX/ma-TZVP method, the single-point energies of reactants and TSs were refined using the DLPNO–CCSD­(T)/CBS­(T-Q) method to examine the performance of the applied DFT method in describing the current reaction systems. In our previous work on the H-abstraction reactions from several small methyl esters initiated by OH radicals, the DLPNO–CCSD­(T)/CBS method was tested, with an average uncertainty of 0.5 kcal mol^–1^ against the gold-standard CCSD­(T)/CBS method,^50^ indicating its ability as a benchmark for assessing the performance of the M08-HX/ma-TZVP method. Table 1 lists the numbers of effective torsions and identified distinguishable conformers of reactants and TSs, and the barrier heights with the zero-point energy (ZPE) correction for each elementary reaction determined by the M08-HX/ma-TZVP and DLPNO–CCSD­(T)/CBS­(T-Q) methods are also presented.

**Table 1:** Numbers of Effective Torsions (*t*) and Identified Distinguishable Conformers (*N*_conformer_) of Reactants and TSs, As Well As Determined Barrier Heights with ZPE Corrections Based on the Most Stable Conformers of Species Using the M08-HX/ma-TZVP (*V*_DFT_^‡^) and DLPNO–CCSD­(T)/CBS­(T-Q)//M08-HX/ma-TZVP (*V*_CC_^‡^) Methods

system	*t*	*N*_conformer_	*V*_DFT_^‡^	*V*_CC_^‡^	system	*t*	*N*_conformer_	*V*_DFT_^‡^	*V*_CC_^‡^
1-butylamine	3	14	0.0	0.0					
TS^1BuA^1–1	3	25	10.2	10.0	TS^1BuA^2–1	4	33	–0.6	0.6
TS^1BuA^1–2	3	16	3.4	3.3	TS^1BuA^2–2	4	22	–1.3	–1.0
TS^1BuA^1–3	3	24	7.3	7.4	TS^1BuA^2–3	4	42	–2.0	–2.4
TS^1BuA^1–4	3	27	6.8	6.5	TS^1BuA^2–4	4	42	–2.6	–3.6
TS^1BuA^1–5	4	37	9.1	10.0	TS^1BuA^2–5	5	68	–1.5	–1.5
2-butylamine	2	9	0.0	0.0					
TS^2BuA^1–1–1	2	9	10.3	10.2	TS^2BuA^2–1–1	3	8	–0.8	0.7
TS^2BuA^1–1–2	2	9	10.5	10.5	TS^2BuA^2–1–2	3	8	–0.9	0.7
TS^2BuA^1–2	2	7	2.6	2.9	TS^2BuA^2–2	3	9	–1.6	–0.7
TS^2BuA^1–3–1	2	9	7.7	8.1	TS^2BuA^2–3–1	3	16	–1.9	–1.5
TS^2BuA^1–3–2	2	9	7.4	7.5	TS^2BuA^2–3–2	3	16	–2.2	–1.7
TS^2BuA^1–4	3	27	9.5	10.5	TS^2BuA^2–4	4	44	–0.6	–0.3
TS^2BuA^1–5	3	27	9.8	10.4	TS^2BuA^2–5	4	44	–0.2	0.3

For the H-abstraction reactions initiated by H radicals, the barrier heights calculated with the M08-HX/ma-TZVP method exhibit remarkable agreement with those obtained by using the DLPNO–CCSD­(T)/CBS­(T-Q) method. The discrepancies between these two methods range from 0.1 to 0.9 kcal mol^–1^ for the reactions 1BuA + H and 0.0–1.0 kcal mol^–1^ for the reactions 2BuA + H. For the H-abstraction reactions initiated by OH radicals, the deviations between these two methods are slightly larger but are still acceptable. The discrepancies range from 0.0 to 1.2 kcal mol^–1^ and 0.3 to 1.6 kcal mol^–1^ for the reactions 1BuA + OH and 2BuA + OH, respectively. Based on these analyses, the average uncertainty is <0.5 kcal mol^–1^, indicating the ability of the M08-HX/ma-TZVP method for conformational analysis and thermal rate constant calculations.

Figure 2 displays the numbers of identified distinguishable conformers of reactants and TSs for the H-abstraction reactions from 1BuA/2BuA initiated by H and OH radicals. Generally, the reaction systems involving 1BuA exhibit more distinguishable conformers due to more effective torsions, particularly in reactions with OH radicals. For the H-abstraction reactions initiated by H radicals, note that the conformers with relative energies <2 kcal mol^–1^ are the majority, and these low-energy conformers play crucial roles in the partition functions, especially at low temperatures. For the H-abstraction reactions initiated by OH radicals, such as TS^1BuA^2–3 to TS^1BuA^2–5 and TS^2BuA^2–3 to TS^2BuA^2–5, an opposite observation is made that the conformers with relative energies higher than 2 kcal mol^–1^ comprise the majority. The reason is that the hydrogen bond interaction between reactants leads to a more stable conformer with a much lower energy. With increasing temperature, these high-energy conformers could significantly promote the multistructural torsional anharmonicity of corresponding TSs.

**Figure 2:**
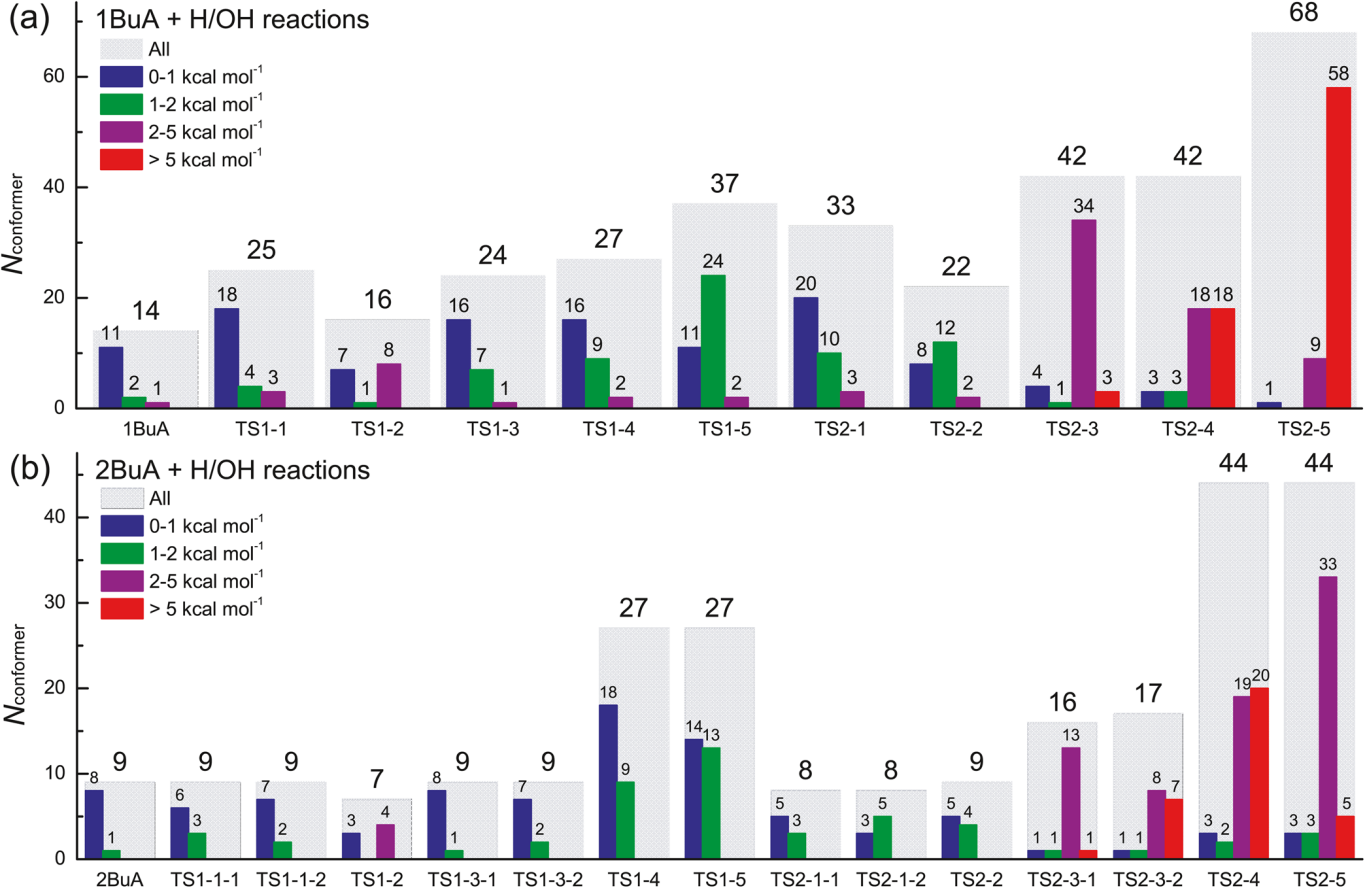
Numbers of identified distinguishable conformers of reactants and TSs for the reactions (a) 1BuA + H/OH and (b) 2BuA + H/OH at the M08-HX/ma-TZVP level of theory.

The MS-T factors of reactants and TSs for the H-abstraction reactions from 1BuA/2BuA initiated by H and OH radicals are shown in Figure 3. For the reactions 1BuA + H, the multistructural torsional anharmonicity of TS^1BuA^1–5 is the most pronounced, and its MS-T factor increases dramatically with increasing temperature below 1400 K. This behavior can be associated with a large number of conformers of this transition state. TS^1BuA^1–1 and TS^1BuA^1–3 also exhibit a significant multistructural torsional anharmonicity with the maximum MS-T factors of around 86 and 55, respectively. The MS-T factors of TS^1BuA^1–2 and TS^1BuA^1–4 are <12 and 8, respectively, indicating their less important multistructural torsional anharmonicity in comparison with other TSs. For the reactions 1BuA + OH, TS^1BuA^2–1 possesses the largest MS-T factor below 800 K. With further increasing temperature, the multistructural torsional anharmonicity of TS^1BuA^2–5 becomes the most remarkable with a maximum MS-T factor of 1240 at 2000 K. This observation is consistent with the fact that TS^1BuA^2–5 has plenty of conformers with relative energy higher than 5 kcal mol^–1^ as aforementioned. Except for the TS^1BuA^1–5 and TS^1BuA^2–5 at the −CH_3_ group, note that all of the TSs at the −CH_2_– and –NH_2_ groups feature a chiral atom due to the attack of H/OH radicals. This characteristic implies that the *R* structures possess corresponding *S* enantiomers that cannot interconvert through simple rotations of the dihedral angles. More precisely, a geometry defined by dihedral angles ϕ_*i*_ in the *R* conformer corresponds to one with angles 360° – ϕ_*i*_ in the *S* enantiomer. Therefore, the statistical weight of these transition states (TSs) is set to 1 in the MS-T factor calculations. Additionally, because the hydrogen atoms bound to the −CH_2_– and –NH_2_ groups are chemically equivalent, the calculated rate constants are multiplied by two to account for both reactive sites.

**Figure 3:**
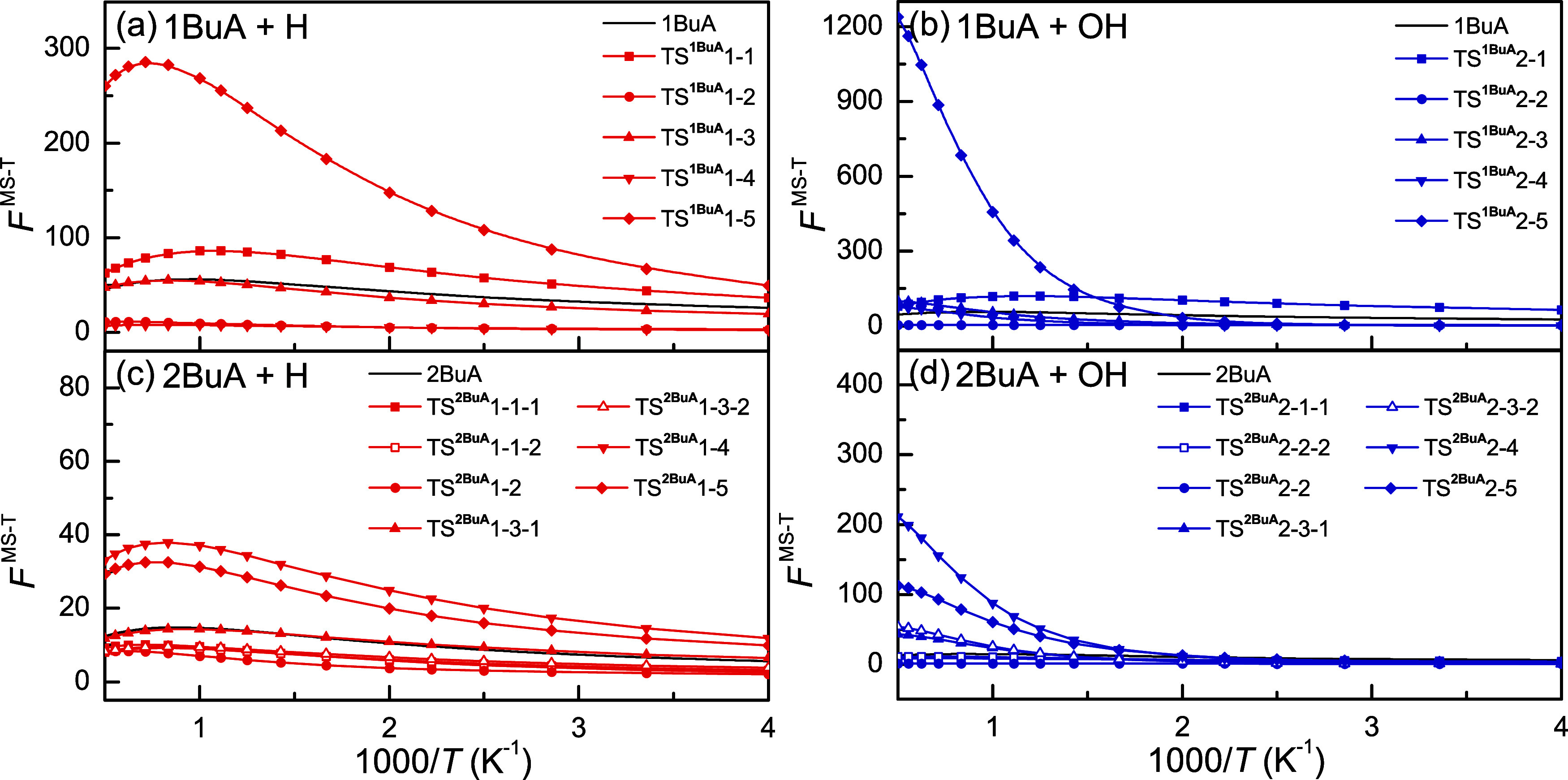
Calculated MS-T factors of reactants and TSs as a function of inverse temperature for reactions (a, b) 1BuA + H/OH and (c, d) 2BuA + H/OH at the M08-HX/ma-TZVP level of theory.

Compared with the 1BuA reaction system, the 2BuA reaction system demonstrates less significant multistructural torsional anharmonicity because of fewer distinguishable conformers. For the reactions 2BuA + H, the reaction channels at the methyl groups (TS^2BuA^1–4 and TS^2BuA^1–5) possess the most pronounced multistructural torsional anharmonicity, and the maximums of their MS-T factors reach 38 and 33, respectively. For the reactions 2BuA + OH, similar observations could be made. It is also important to note that the weight of 2BuA and all of the TSs should be set to 1 due to the presence of the chiral carbon atom. In addition, two H atoms attached to the –NH_2_ or −CH_2_– groups possess different chemical environments, which should be treated as two distinct reaction channels. As mentioned above, only the *R* structure of 2BuA was considered for kinetic calculations, and the final calculated rate constants should be further doubled to include the possibility of its *S* enantiomer.

### Potential Energy Profile

Figure 4 summarizes the potential energy profiles for the H-abstraction reactions from 1BuA/2BuA initiated by H and OH radicals, determined at the M08-HX/ma-TZVP level of theory. The Cartesian coordinates and nonscaled vibrational frequencies for these most stable stationary points are listed in Section S1 of the Supporting Information. For reaction 1BuA + H, the relative energies of TSs range from 3.4 to 10.2 kcal mol^–1^. The reaction channel at the N-site (R^1BuA^1–1) has the highest barrier height with a TS energy of 10.2 kcal mol^–1^ due to the strong N–H bond, followed by the reaction channel at the C_δ_-site (R^1BuA^1–5) with a TS energy of 9.1 kcal mol^–1^. The hydrogen atom at the C_α_-site (R^1BuA^1–2) possesses the lowest barrier height (3.4 kcal mol^–1^) as expected because of the –NH_2_ group, which weakens the adjacent C–H bond energy. For the reactions 2BuA + H, similar observations could be made that the reaction channel at the C_α_-site (R^2BuA^1–2) has the lowest barrier height, while the reaction channels at the primary C and N atoms exhibit high barrier heights. These features are mainly determined by the strength of the bond dissociation energy of the C–H and N–H bonds. Note that the TS energies at the N-site are 10.3 and 10.5 kcal mol^–1^, respectively, indicating that the abstracted H atoms have different chemical environments. Similar observations between R^2BuA^1–3–1 and R^2BuA^1–3–2 also support the conclusion.

**Figure 4:**
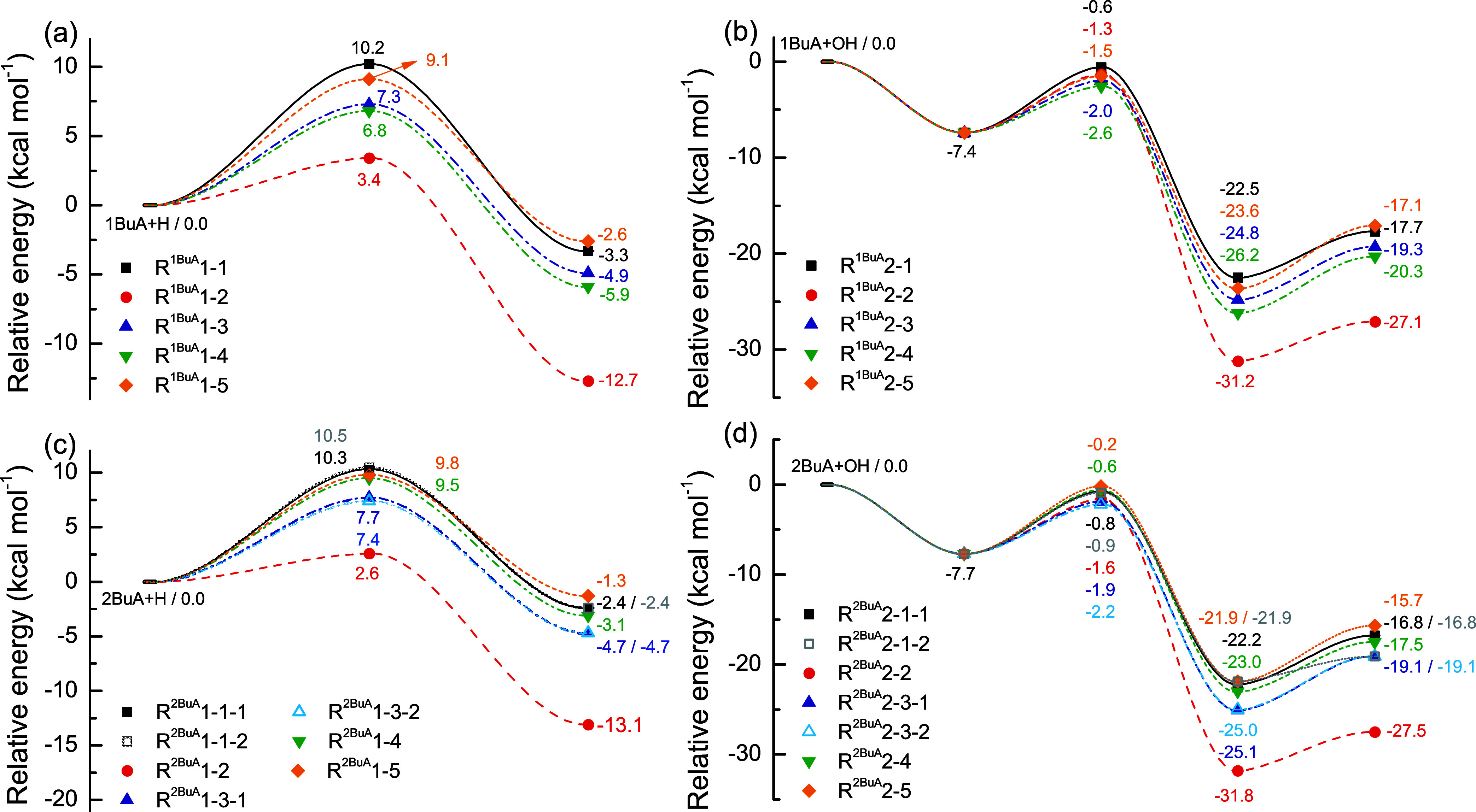
Potential energy profiles for the reactions (a, b) 1BuA + H/OH and (c, d) 2BuA + H/OH at the M08-HX/ma-TZVP level of theory based on the most stable conformers of each species.

For the H-abstraction reactions from 1BuA/2BuA initiated by OH radicals, the RCs are first identified in the entrance process, and their energies are −7.4 and −7.7 kcal mol^–1^ lower than those of reactants, respectively, due to the strong hydrogen bond interactions. For the reactions 1BuA + OH, the reaction channel at the N-site (R^1BuA^2–1) has the highest barrier height with a TS energy of −0.6 kcal mol^–1^, followed by the channel at the C_α_-site. This observation contrasts significantly with the case of the reactions 1BuA + H, and the reason could be attributed to the considerable steric hindrance between the –NH_2_ group and OH radical. This hindrance prevents the OH radical from effectively attacking the H atom at the C_α_-site. Hence, the reaction channels at the methylene sites (R^1BuA^2–3 and R^1BuA^2–4) have the lowest barrier heights. For the reactions 2BuA + OH, the reaction channels at the methyl groups (R^2BuA^2–4 and R^2BuA^2–5) exhibit the highest barrier heights due to the strong C–H bond, followed by the channel at the N-site (R^2BuA^2–1) and the C_α_-site (R^2BuA^2–2).

To explore the effects of the –NH_2_ group and the alkyl chain on the barrier heights at different reaction sites, we compared the relative energies of RCs and TSs for the H-abstraction reactions initiated by H and OH radicals from a series of C1–C4 amines, including methylamine (MA),^16^ ethylamine (EA),^16^ *n*-propylamine (NPA),^22^ *iso*-propylamine (IPA),^23^ 1BuA,^25^ and 2BuA. For the H-abstraction reactions initiated by H radicals in Figure 5a, the relative energies of the TSs at the N-site are around 10.0 kcal mol^–1^, and the values decrease to ∼3.6 kcal mol^–1^ at the C_α_-site due to the influence of the –NH_2_ group, except for MA as it involves the −CH_3_ rather than the −CH_2_– group. At the C_β_-site, the TSs involving the −CH_2_– group have an average energy of 7.5 kcal mol^–1^, and their energies gradually decrease and are close to 7.0 kcal mol^–1^ with further increasing distance between the –NH_2_ group and reaction site. The reason is that there is a weak intramolecular interaction between the N atom of –NH_2_ group and the H atom of C_γ_-site in the TS structures, and the attack of the H radical on the C_β_-site results in a certain steric effect, thus leading to a slightly higher TS energy by 0.5 kcal mol^–1^. For the TSs involving the −CH_3_ group, a similar observation is made that their energies are gradually closer to 10.0 kcal mol^–1^ with an increasing length of the alkyl chain. To eliminate the influence of the NH_2_ group, the TS energies of the reactions *n*-decane + H at the terminal −CH_3_ group and its adjacent −CH_2_– group were adopted for comparison, which reflects the trend of the TS energy.

**Figure 5:**
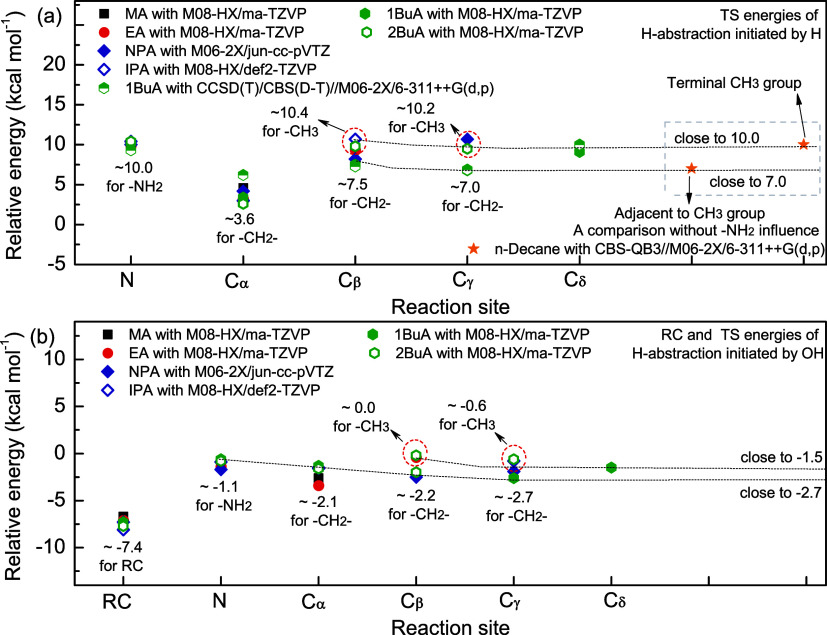
Relative energies of RCs and TSs as a function of reaction sites for the H-abstraction reactions initiated by (a) H and (b) OH radicals from MA,^16^ EA,^16^ NPA,^22^ IPA,^23^ 1BuA,^25^ 2BuA, and *n*-decane.^51^

For the H-abstraction reactions initiated by OH radicals in Figure 5b, the relative energies of RCs are around 7.4 kcal mol^–1^ lower than that of reactants because of the strong hydrogen bond interactions between the N atom of the NH_2_ group and the H atom of the OH radical. The TSs at the N-site have an average energy of ∼ −1.1 kcal mol^–1^. For the TSs involving the −CH_2_– group at the C_α_-site, their energies are only around −2.1 kcal mol^–1^, which is lower than expected, as the effect of the –NH_2_ group should significantly reduce the bond dissociation energy of the C_α_–H bond. The reason is that the attack of the OH radical on the C_α_-site leads to a considerable steric effect due to the repulsion effect. With increasing distance between the –NH_2_ group and the reaction site, the relative energies of TSs involving the −CH_2_– group gradually decrease and are close to −2.7 kcal mol^–1^, while the relative energies of TSs at the terminal −CH_3_ group are gradually close to −1.5 kcal mol^–1^.

### Variational and Tunneling Effects

The influences of the variational effect on the rate constants are quantified and are shown in Figure 6. For the H-abstraction reactions initiated by H radicals, the recrossing coefficients for the reaction channels involving the −CH_2_– group are approximately 1 within the investigated temperature range, such as R^1BuA^1–3, R^1BuA^1–4, R^2BuA^1–3–1, and R^2BuA^1–3–2, indicating their negligible variational effects. In comparison with those of these channels, the variational effects of the channels at other reaction sites are more pronounced, leading to a significant reduction in the rate constants at low temperatures, particularly for the channels at the C_α_-site. For the H-abstraction reactions initiated by OH radicals, the variational effects in this reaction system are more remarkable, with recrossing coefficients decreasing as the temperature decreases. Except for R^1BuA^2–1, R^1BuA^2–2, R^2BuA^2–1–1, and R^2BuA^2–1–2, the recrossing coefficients of other reaction channels are all <0.5.

**Figure 6:**
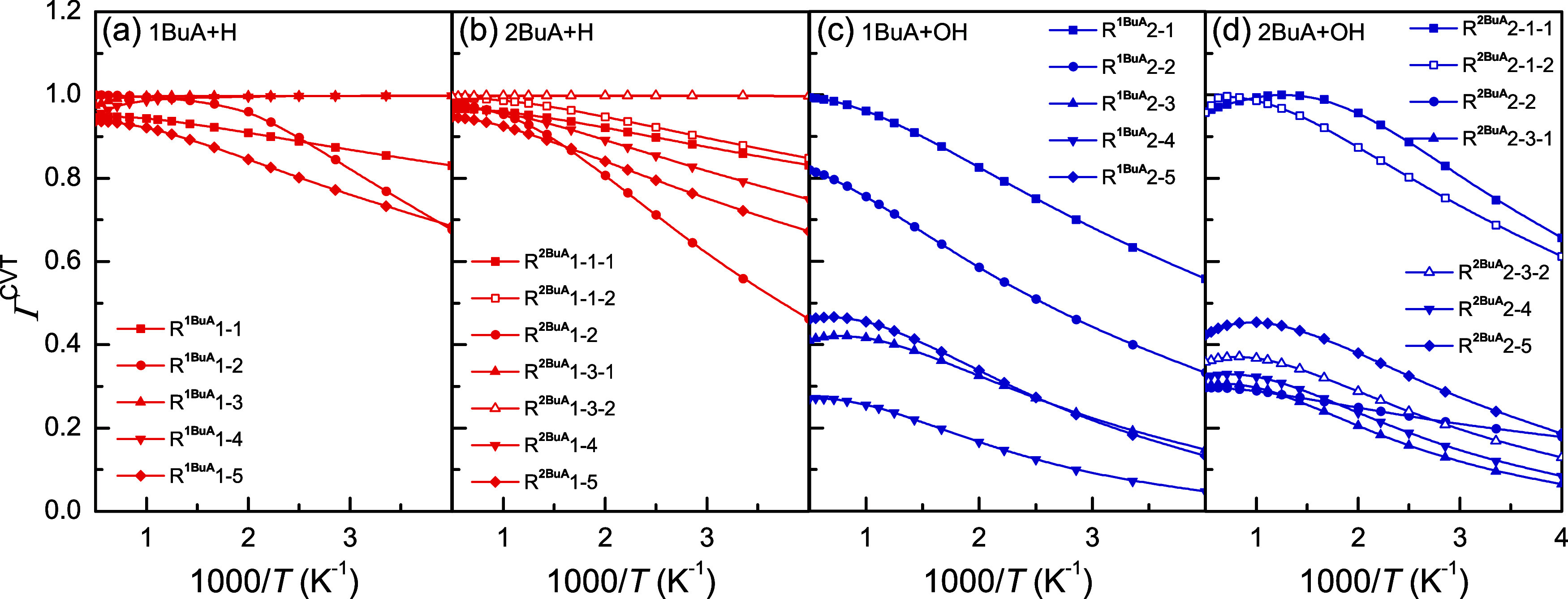
Recrossing coefficients as a function of inverse temperature for the H-abstraction reactions from 1BuA/2BuA initiated by (a, b) H radicals and (c, d) OH radicals.

Figure 7 displays the tunneling coefficients for the H-abstraction reactions from 1BuA/2BuA initiated by H and OH radicals. Generally, the tunneling effect is more significant at low temperatures, and the tunneling coefficients of the H-abstraction reactions initiated by H radicals are higher than those of reactions initiated by OH radicals due to the much higher barrier heights. In addition, it is important to note that the recrossing and tunneling coefficients for the two reaction channels at the N- or C_β_-sites in the reactions 2BuA + H/OH are different, suggesting that these two channels must be considered separately.

**Figure 7:**
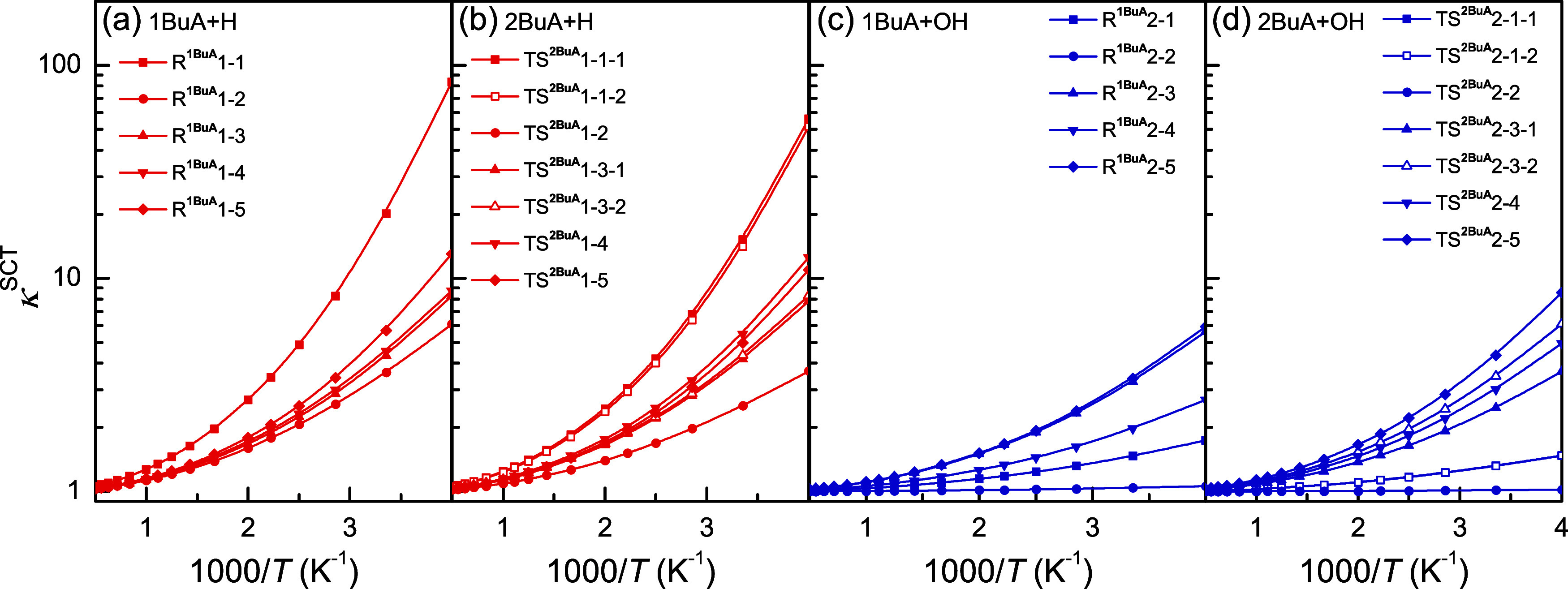
Tunneling coefficients as a function of inverse temperature for the H-abstraction reactions from 1BuA/2BuA initiated by (a) H radicals and (c) OH radicals.

### Rate Constants and Branching Ratios

Figure 8 shows the calculated rate constants and branching ratios for the H-abstraction reactions from 1BuA initiated by H and OH radicals. The corresponding rate constant values are provided in Section S2 of the Supporting Information. These data were fitted using the four-parameter analytical expression proposed by Zheng and Truhlar,^52^ as detailed in Section S3. For the reactions 1BuA + H in Figure 8a,b, it is evident that the reaction channel at the C_α_-site (R^1BuA^1–2) is dominant with a branching ratio larger than 0.38 at 250–2000 K. The reaction channel at the C_β_-site (R^1BuA^1–3) is the second most important one, followed by R^1BuA^1–4, and their branching ratios gradually increase with increasing temperature. At high temperatures, nevertheless, the reaction channel at the terminal −CH_3_ group (R^1BuA^1–5) becomes more important than R^1BuA^1–4, probably due to its significant multistructural torsional anharmonicity. For the reactions 1BuA + OH in Figure 8c,d, an interesting observation is made that the reaction channel at the N-site (R^1BuA^2–1) with the highest barrier height exhibits the highest rate constants over the investigated temperature range. The reason can be attributed to the fact that other reaction channels have significant variation effects. The reaction channel at the terminal −CH_3_ group (R^1BuA^2–5) is the second most important one with a branching ratio of around 0.3 at 250–2000 K because of its significant multistructural torsional anharmonicity.

**Figure 8:**
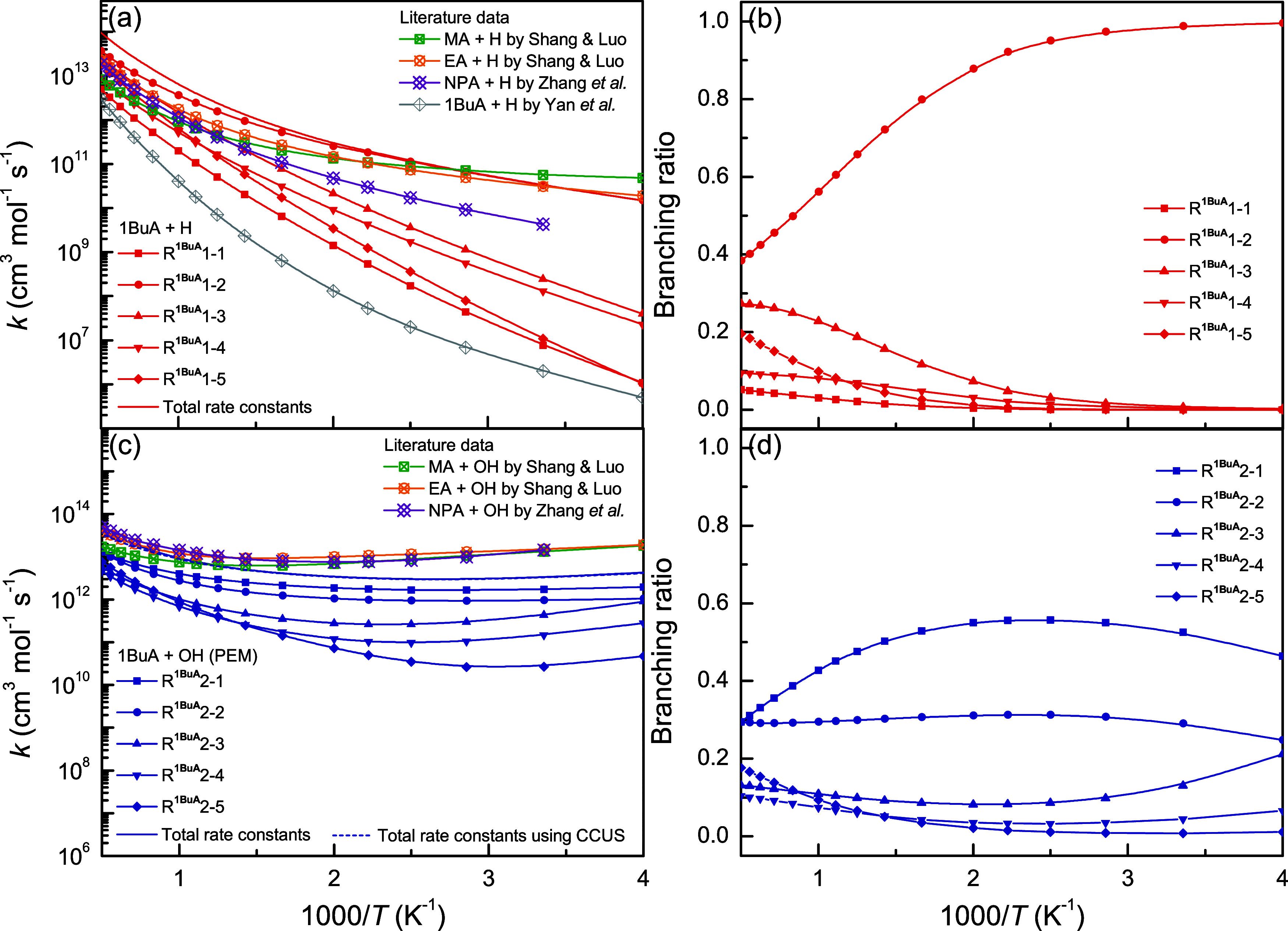
Calculated rate constants and branching ratios as a function of inverse temperature for the reactions (a, b) 1BuA + H and (c, d) 1BuA + OH, as well as the comparison with the available literature data.^16,22,25^

The reported rate constants for the H-abstraction reactions from MA, EA, NPA, and 1BuA initiated by H and OH radicals in the literature were also adopted for comparison.^16,22,25^ Our calculated rate constants for the reactions 1BuA + H agree well with those of the reactions MA/EA + H but are higher than those of the reactions NPA + H by 1 order of magnitude, probably due to the difference in the applied DFT methods. Note that our calculations are significantly higher than the reported data from Yan et al.^25^ by 2–5 orders of magnitude, and this substantial discrepancy may be attributed to the neglect of the multistructural torsional anharmonicity. At 1000 K, for example, the calculated multistructural torsional factors for R^1BuA^1–1 to R^2BuA^1–5 range from 0.17 to 4.79, reflecting on the overall rate coefficients obtained. For the H-abstraction reactions initiated by OH radicals, our calculations are in line with the rate constants of the reactions MA/EA/NPA + OH at high temperatures but are approximately 3 times lower at low temperatures, which falls within the acceptable uncertainty.

Figure 9 shows the calculated rate constants and branching ratios for the H-abstraction reactions from 2BuA initiated by H and OH radicals. For the reactions 2BuA + H in Figure 9a,b (see Sections S2 and S3), the reaction channel at the C_α_-site (R^2BuA^1–2) dominates and its branching ratio gradually decreases from 0.99 at 250 K to 0.34 at 2000 K. The reaction channels at the −CH_2_– and −CH_3_ groups compete with each other, and their branching ratios increase from 0.0 to ∼0.2 with increasing temperature. The branching ratios of R^2BuA^1–1–1 and R^2BuA^1–1–2 are similar, and both are no more than 0.03, indicating their minor importance. For the reactions 2BuA + OH, an opposite observation is made that the reaction channels at the N-site (R^2BuA^2–1–1 and R^2BuA^2–1–2) are important and compete with the channel at the C_α_-site (R^2BuA^2–2) due to their insignificant variational effects.

**Figure 9:**
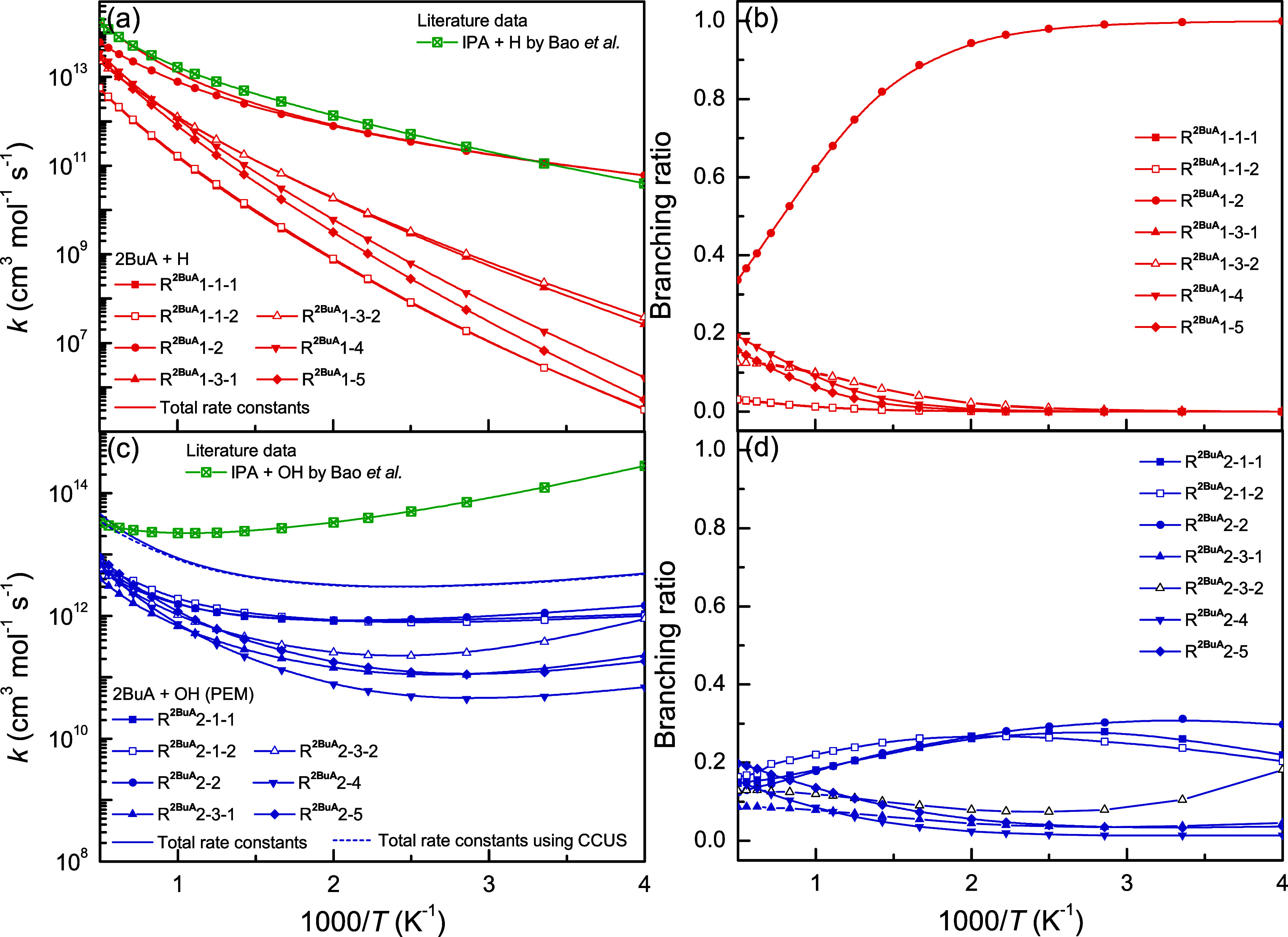
Calculated rate constants and branching ratios as a function of inverse temperature for the reactions (a, b) 2BuA + H and (c, d) 2BuA + OH, as well as the comparison with the available literature data.^23^

In addition, our calculated rate constants for the reactions 2BuA + H/OH were also compared with its homologue reactions, IPA + H/OH.^23^ For the H-abstraction reactions initiated by H radicals, good agreement is achieved between these two reaction systems. For the H-abstraction reactions initiated by OH radicals, nevertheless, our calculations are much lower than the rate constants for the reactions IPA + OH by around 60 times. During the kinetic calculations, the competitive canonical unified statistical theory (CCUS) method^33,53,54^ was also applied to determine the accurate rate constants (for more details, see Section S4 of the Supporting Information). As shown in Figures 8 and 9, the calculations by the PEM and CCUS methods are consistent with each other, indicating the reliability of our calculations.

## Conclusions

In this study, we employed the multistructural canonical variational theory with small-curvature tunneling (MS-CVT/SCT) to compute accurate constants for the H-abstraction reactions from 1-butylamine and its isomer, 2-butylamine, by two key active radicals (H and OH) during fuel combustion. Given the chiral structure of 2-butylamine, special attention was devoted to thoroughly considering all of the possible reaction channels. The M08-HX/ma-TZVP method was applied for geometry optimization and kinetic calculation, with the average uncertainty <0.5 kcal mol^–1^ compared to the high-level DLPNO–CCSD­(T) method. For the OH-initiated reactions, the pre-equilibrium model (PEM) and the competitive canonical unified statistical (CCUS) theory were both adopted, and a good agreement suggests the reliability of the applied methodologies.

The influence of the position of the amino functional group on the kinetics was explored, and the comparison shows that the 1-butylamine reaction system exhibits more pronounced multistructural torsional anharmonicity. Generally, the H-abstraction reaction at the N-site exhibits the highest barrier height, and the barrier height gradually decreases with increasing distance between the amino functional group and reaction site. The kinetic calculations demonstrate that the reaction channel at the C_α_-site is dominant for the reactions with H radicals due to its lowest barrier height. For the OH-initiated reactions, nevertheless, the reaction channel at the N-site rather than C_α_-site shows more proportion due to its more significant multistructural torsional anharmonicity and less important variational effect. These findings and kinetic data provide comprehensive insight into the oxidation mechanism of C4 amines and are expected to guide further theoretical and experimental investigation concerning their combustion behavior.

## Supplementary Material



## Notes

Representation of dihedral angles used in conformational search; Cartesian coordinates and nonscaled vibrational frequencies for the most stable conformers of reactants and transition states; rate constants for all reactions; CCUS formalism; and fit parameters of rate constants (PDF)
