# Supplementary material for: Comprehensive Oxidation Mechanism of n‑Butylamine and 2‑Butylamine by H and OH Radicals: Insights into Reactivity
Source: J Phys Chem A. 2025 Jun 2;129(23):5107–17. doi: 10.1021/acs.jpca.5c02556 (PMC12169691; doi:10.1021/acs.jpca.5c02556)
Supplement: Supplementary file 1 [file jp5c02556_si_001.pdf]

Supporting Information

Comprehensive Oxidation Mechanism of *n*-Butylamine and 2-Butylamine by H and OH Radicals: Insights into Reactivity

Joel Leitão Nascimento[†], Tiago Vinicius Alves[*,†], and Yanlei Shang[*,‡,¶]

[†]*Departamento de Físico-Química, Instituto de Química, Universidade Federal da Bahia, Rua Barão de Jeremoabo, 147, 40170-115- Salvador, Bahia, Brazil*
[‡]*Energy Research Institute, Qilu University of Technology (Shandong Academy of Sciences), Jinan, Shandong, 250014, P. R. China*
[¶]*School of Materials Science and Engineering, Southwest Jiaotong University, Chengdu, Sichuan, 610031, P. R. China*

*E-mail address: tiagova@ufba.br; ylshang@sderi.cn

Sections

S1. Cartesian Coordinates and Noncaled Vibrational Frequencies S3

S1.1 1BuA + H/OH S3

S1.2 2BuA + H/OH S9

S2. Rate Constants - 1BuA + H/OH S17

S3. Rate Constants - 2BuA + H/OH S18

S4. Fit of rate constants S19

S5. CCUS Rate Constant S20

Tables

S1. Fitting Parameters to the PEM//MS-CVT/SCT rate constant at 250-2000 K. S20

Figures

S1. Representation of dihedral angles used in the conformational search. Carbon, nitrogen, and oxygen are represented by gray, blue, and red, respectively. Dihedrals of methyl groups, represented by red arrows, are not included in the conformational search. S17

S1. Cartesian Coordinates and Noncaled Vibrational Frequencies

S1.1 1BuA + H/OH

Geometry – 1BuA – C_1				Frequency (cm^{-1})
C	-1.73165700	-0.73915400	0.30714300	94.28 178.51 247.73 264.37 304.48 380.40
C	-1.11719300	0.32357600	-0.59661000	523.25 727.37 805.42 850.60 876.28 947.14
C	0.03188000	1.07784600	0.07392500	984.04 1038.27 1061.22 1127.32 1139.04 1202.41
C	1.13039300	0.16647400	0.61232300	1267.96 1310.77 1315.18 1362.93 1387.79 1398.73
N	1.61494000	-0.72498600	-0.43590900	1420.83 1465.99 1473.29 1483.16 1492.12 1516.64
H	-2.63084700	-1.17293100	-0.14136500	1651.48 2973.07 3012.05 3028.62 3039.70 3049.99
H	-1.02219900	-1.55528800	0.48148900	3064.12 3086.07 3108.09 3120.33 3537.72 3627.16
H	-2.01234500	-0.31783100	1.28091900	
H	-1.88385500	1.04741300	-0.90032800	
H	-0.75067400	-0.15455900	-1.51222900	
H	-0.35585800	1.68055300	0.90686000	
H	0.47219100	1.78718000	-0.64196000	
H	0.72449900	-0.45665100	1.41961300	
H	1.92150300	0.78843600	1.06344700	
H	2.02179500	-0.19375900	-1.19760200	
H	2.33066800	-1.35011800	-0.08816300	

Geometry – TS^{1BuA}1-1 – C_1				Frequency (cm^{-1})
C	-1.82958100	0.60441100	0.42256300	-1564.03
C	-1.11490800	-0.21437200	-0.64777400	135.82 141.78 213.11 275.08 290.78 392.31
C	-0.00913300	-1.10552000	-0.08279600	415.31 499.39 708.75 740.32 811.69 862.35
C	1.11965000	-0.33079800	0.58526500	932.48 958.88 1018.05 1053.78 1076.77 1108.18
N	1.80592900	0.52554400	-0.37326100	1141.56 1187.98 1226.88 1272.12 1315.94 1356.53
H	-2.66569900	1.16974800	-0.00062900	1376.69 1383.69 1401.88 1423.08 1455.68 1465.50
H	-2.22788400	-0.04481900	1.21191600	1479.22 1489.51 1496.70 1744.95 2991.01 3022.95
H	-1.15536700	1.32823200	0.89512800	3027.64 3035.26 3039.64 3074.82 3082.56 3102.96
H	-0.69114600	0.45267000	-1.40983000	3117.30 3476.34
H	-1.84282800	-0.84741000	-1.16968300	
H	0.42209200	-1.71218800	-0.88856700	
H	-0.44089000	-1.79964200	0.65172600	
H	1.86637300	-1.03433400	0.98240100	
H	0.74442800	0.23166800	1.45580600	
H	1.10325400	1.55373800	-0.52577900	
H	2.60901800	0.95534400	0.09036200	
H	0.64098100	2.34585600	-0.34357300	

Geometry – TS^{1BuA}1-2 – C_1				Frequency (cm^{-1})
C	2.54677400	-0.28055100	0.09853600	-1544.24
C	1.25283500	0.47441400	-0.17979000	91.51 115.31 178.80 231.07 284.27 296.93
C	0.01927000	-0.39364600	0.03994300	330.67 403.17 440.79 732.44 772.13 824.65
C	-1.26985700	0.35652000	-0.21316400	904.51 955.32 1000.74 1066.66 1095.05 1154.62
N	-2.43210200	-0.44256400	-0.01272800	1170.28 1218.40 1247.17 1285.27 1307.84 1314.29
H	3.42648200	0.34879300	-0.06725700	1343.60 1386.55 1393.69 1401.79 1436.69 1469.93
H	2.57773400	-0.63248800	1.13609100	1480.52 1484.67 1496.00 1654.42 3002.74 3026.01
H	2.63523100	-1.15912300	-0.55078400	3033.88 3041.96 3061.38 3071.94 3109.46 3119.48
H	1.25226000	0.84479000	-1.21407100	3559.84 3655.68

H	1.18972200	1.36130900	0.46485200	
H	0.01647600	-0.77174100	1.07421500	
H	0.05363500	-1.27790600	-0.61286300	
H	-1.28994900	0.81864200	-1.20785700	
H	-1.23075700	1.31404800	0.54738500	
H	-2.46089500	-0.86151800	0.90798600	
H	-3.29520000	0.05538100	-0.18065100	
H	-1.14415600	2.11733700	1.31889600	
Geometry – TS^{1BuA}1-3 – C_1				Frequency (cm^{-1})
C	1.82341400	-0.73417700	0.13957300	-1546.26
C	1.10834400	0.45774800	-0.49677500	93.94 165.71 229.51 242.61 276.29 290.98
C	-0.05229300	0.93849700	0.34692100	318.57 435.93 542.10 751.29 820.97 853.57
C	-1.11504400	-0.08433100	0.67630600	900.39 952.70 989.80 1042.32 1071.83 1120.64
N	-1.52931100	-0.78443000	-0.53279400	1143.69 1180.66 1249.83 1254.03 1269.70 1311.72
H	2.70902100	-1.01635300	-0.43837000	1335.78 1383.11 1393.22 1410.37 1417.62 1470.13
H	2.14674100	-0.50524100	1.16209800	1481.09 1486.46 1503.82 1652.82 2974.89 3023.99
H	1.15613600	-1.60188200	0.17621300	3031.76 3046.56 3082.88 3096.61 3113.40 3122.06
H	0.73958700	0.16312300	-1.48608400	3534.61 3628.93
H	1.81117500	1.28588200	-0.64711100	
H	-0.66728000	1.80498600	-0.35650000	
H	0.25339400	1.51073000	1.22918900	
H	-1.94132700	0.41886000	1.20630300	
H	-0.70126200	-0.82629300	1.37586800	
H	-2.23919400	-1.47723800	-0.33346200	
H	-1.92226600	-0.12868000	-1.20001100	
H	-1.22607700	2.39669500	-0.95472800	
Geometry – TS^{1BuA}1-4 – C_1				Frequency (cm^{-1})
C	-2.36728000	-0.28815300	-0.16875000	-1560.76
C	-0.94944900	-0.21284800	0.33829700	83.94 149.11 208.90 213.23 261.85 298.18
C	0.01438000	0.56178000	-0.52771800	321.71 347.97 504.47 781.75 829.52 857.22
C	1.42754500	0.58325500	0.04430700	881.93 968.66 974.28 1043.19 1084.91 1128.45
N	1.98367600	-0.76602500	0.06391800	1143.07 1182.64 1242.14 1263.71 1277.68 1305.88
H	-3.02804500	-0.78892000	0.54546200	1328.38 1391.99 1402.89 1405.40 1415.71 1458.90
H	-2.77054700	0.71320100	-0.36068100	1477.24 1482.63 1498.44 1653.40 2979.17 2985.55
H	-2.41315200	-0.84698400	-1.11325300	3018.58 3050.88 3060.71 3085.09 3107.53 3120.92
H	-0.53495100	-1.17244800	0.65948800	3543.73 3632.88
H	-0.99553300	0.44788300	1.41929300	
H	-0.34792000	1.59149000	-0.65504300	
H	0.03834000	0.11902100	-1.53811600	
H	1.38213700	0.94442500	1.08028800	
H	2.04183000	1.30362300	-0.51994400	
H	2.17694100	-1.08743400	-0.87755800	
H	2.85336400	-0.80064000	0.57963900	
H	-1.03937300	1.07475500	2.21618200	
Geometry – TS^{1BuA}1-5 – C_1				Frequency (cm^{-1})
C	-1.79850500	0.58933700	0.27664200	-1168.78

C	-1.08838400	-0.41100900	-0.60042300	137.92 146.80 219.80 271.54 287.21 369.05
C	0.06587000	-1.12579700	0.10214400	404.56 489.58 571.34 758.53 821.85 854.92
C	1.15247300	-0.17902200	0.60015800	889.17 944.32 985.91 1032.79 1071.50 1127.66
N	1.73432600	0.56693400	-0.51054200	1151.45 1163.41 1227.05 1232.06 1258.08 1281.02
H	-1.02843200	1.64491500	0.39906900	1342.09 1362.83 1384.99 1422.27 1443.70 1463.40
H	-2.69898300	1.03269800	-0.15160800	1474.55 1499.72 1537.40 1659.85 2969.61 3014.24
H	-1.94682000	0.27731600	1.31378200	3026.90 3052.62 3070.80 3082.43 3086.36 3168.43
H	-0.72324500	0.08167300	-1.51255700	3535.37 3620.58
H	-1.81456700	-1.16079600	-0.94684900	
H	0.51983800	-1.84474500	-0.59008300	
H	-0.33013400	-1.69338500	0.95572400	
H	1.95021800	-0.77009200	1.06464100	
H	0.74124900	0.47247200	1.39162400	
H	1.08721600	1.27464100	-0.84152900	
H	2.56988600	1.05621400	-0.21531600	
H	-0.45523000	2.41950100	0.43577300	
Geometry – RC^{1BuA}2 – C$_1$				Frequency (cm^{-1})
C	2.56503800	0.39667300	0.17185300	40.68 89.98 114.01 131.81 230.87 238.12
C	1.05314000	0.32818400	-0.00689000	261.43 301.93 346.78 496.12 631.41 714.69
C	0.55971400	-1.10841700	-0.15365600	749.84 827.72 861.84 915.17 967.50 992.42
C	-0.92426300	-1.22000300	-0.47406300	1054.18 1069.07 1126.72 1152.13 1219.39 1279.37
N	-1.73232000	-0.58582200	0.57389100	1294.95 1327.26 1333.59 1400.51 1401.45 1421.59
H	2.91063300	1.42832800	0.28661400	1468.54 1482.60 1485.88 1499.53 1503.24 1644.16
H	2.88020400	-0.16293200	1.06054800	2998.60 3003.97 3024.57 3031.87 3049.07 3055.67
H	3.08248600	-0.03718600	-0.69167300	3072.44 3105.23 3122.39 3471.42 3530.75 3615.21
H	0.75113800	0.90487800	-0.89290000	
H	0.56062300	0.80576200	0.84970600	
H	0.77706800	-1.66854900	0.76945300	
H	1.12498700	-1.60876900	-0.95144300	
H	-1.18819200	-2.27750800	-0.62533100	
H	-1.13171000	-0.69341800	-1.41564800	
H	-2.71670200	-0.79049400	0.44544200	
H	-1.47148300	-0.94374300	1.48696500	
O	-1.42743000	2.18480600	-0.21712500	
H	-1.55514700	1.28731500	0.17457000	
Geometry – TS^{1BuA}2-1 – C$_1$				Frequency (cm^{-1})
C	-1.71442800	1.03732000	0.47108600	-655.69
C	-1.33929200	0.08898700	-0.66310400	78.43 120.64 128.19 162.04 188.79 275.04
C	-0.79267300	-1.25073500	-0.16754500	283.65 370.14 402.65 482.99 636.03 722.63
C	0.52521500	-1.14386300	0.59069000	742.88 804.70 862.51 934.90 972.89 1043.90
N	1.59628500	-0.70040700	-0.28127400	1078.84 1116.27 1136.80 1198.00 1233.97 1284.13
H	-2.20033700	1.93888900	0.08529900	1323.08 1361.16 1383.75 1402.44 1413.49 1459.90
H	-2.40875300	0.55501300	1.17061000	1465.83 1475.10 1488.42 1501.69 1606.26 2489.65
H	-0.82769300	1.36118400	1.02428200	2996.48 3025.48 3029.77 3037.84 3058.29 3074.80
H	-0.60617500	0.57555800	-1.31953000	3082.41 3110.24 3130.26 3592.69 3837.38
H	-2.22036700	-0.10897400	-1.28614200	

H	-0.64636300	-1.93268400	-1.01456500	
H	-1.53305000	-1.71629600	0.49766700	
H	0.80803400	-2.12818400	0.98609900	
H	0.41456000	-0.47430300	1.45836300	
H	1.48523700	0.28935300	-0.65858600	
H	2.51395400	-0.81462900	0.13438800	
O	1.45741200	1.64574100	-0.10136000	
H	2.31473200	1.80173600	0.31515100	
Geometry – TS^{1BuA}2-2 – C_1				Frequency (cm^{-1})
C	2.80324600	-0.00320900	-0.18884200	-331.01
C	1.39820300	0.27583700	0.33118000	55.25 65.56 107.23 117.35 130.73 182.73
C	0.38434800	-0.75067700	-0.15890200	237.92 296.13 398.47 431.77 539.76 734.20
C	-1.00893700	-0.50349300	0.39165000	778.38 809.89 905.16 942.52 997.89 1067.33
N	-1.96729500	-1.44068000	-0.12480300	1093.53 1145.15 1159.95 1221.41 1257.43 1285.49
H	3.52220100	0.74269900	0.16376600	1305.64 1325.17 1383.70 1402.65 1417.95 1439.69
H	3.15349900	-0.98864900	0.13874600	1471.60 1483.31 1485.54 1498.58 1654.78 2109.71
H	2.82118700	0.00629000	-1.28463400	3011.72 3022.45 3033.87 3043.96 3055.40 3075.48
H	1.07498700	1.27599900	0.01215500	3110.25 3119.95 3557.85 3650.54 3796.70
H	1.40349800	0.28229900	1.43054800	
H	0.70066900	-1.76446400	0.12311100	
H	0.34571400	-0.72933600	-1.25826700	
H	-1.28609300	0.57117000	0.14140800	
H	-1.00364100	-0.56185500	1.48851200	
H	-2.89355400	-1.30106200	0.25550800	
H	-2.02267700	-1.40305300	-1.13503700	
O	-1.54671100	2.16418800	-0.24994400	
H	-1.13219500	2.53047200	0.54684400	
Geometry – TS^{1BuA}2-3 – C_1				Frequency (cm^{-1})
C	2.46091600	-0.07283400	-0.16181000	-1104.28
C	1.08676400	-0.70330500	-0.35433800	73.85 111.20 143.33 196.59 243.46 253.71
C	0.11278600	-0.27020600	0.72242700	298.12 357.15 488.31 572.39 668.93 791.35
C	-1.30279000	-0.78934200	0.56965500	841.05 878.31 924.08 963.23 982.04 1036.10
N	-1.89208700	-0.32917800	-0.68930500	1063.84 1082.72 1130.37 1194.77 1204.95 1229.06
H	3.16103800	-0.38057100	-0.94416100	1289.56 1309.05 1328.06 1396.29 1399.34 1410.02
H	2.37753500	1.01929700	-0.18594600	1464.94 1484.27 1485.27 1491.91 1575.41 1653.99
H	2.88972400	-0.35716300	0.80589100	2966.20 3002.06 3036.67 3051.54 3067.44 3078.38
H	1.16419000	-1.80202200	-0.35416700	3115.38 3125.55 3553.33 3637.32 3683.28
H	0.69385700	-0.41190300	-1.33781900	
H	0.03829800	0.91623300	0.64583900	
H	0.50375600	-0.46247400	1.72971700	
H	-1.91939000	-0.40065600	1.38771300	
H	-1.29770100	-1.88986000	0.66366600	
H	-1.49092900	-0.81751400	-1.48105700	
H	-2.88942700	-0.49993100	-0.70790300	
O	-0.38157600	2.09395000	0.05213100	
H	-1.07979500	1.65333300	-0.46929000	

Geometry – TS^{1BuA}2-4 – C_1				Frequency (cm^{-1})
C	-2.42036000	-0.30363600	-0.17125000	-952.39
C	-1.02439700	-0.18622800	0.40447800	53.85 129.38 138.68 211.05 220.49 246.18
C	-0.01057800	-1.09874100	-0.26104800	274.59 345.37 494.42 641.93 710.98 785.77
C	1.41078500	-0.97335900	0.27972200	824.00 872.82 907.60 978.62 997.30 1041.71
N	2.05091000	0.25653200	-0.19735100	1067.68 1097.16 1126.33 1149.99 1213.22 1286.29
H	-3.11581700	0.38471800	0.31707300	1302.21 1334.26 1342.11 1364.58 1395.85 1409.74
H	-2.41823900	-0.07751400	-1.24320900	1419.20 1457.45 1476.11 1481.93 1499.16 1646.31
H	-2.80860800	-1.32327900	-0.04556700	2994.30 3006.66 3023.71 3042.69 3057.12 3068.13
H	-1.01617300	-0.32188100	1.49386100	3097.82 3125.71 3532.48 3616.19 3618.45
H	-0.70043500	0.94091100	0.26143900	
H	-0.00436300	-0.91310400	-1.34588700	
H	-0.34482100	-2.13937100	-0.13404400	
H	1.98593200	-1.87288200	0.01175000	
H	1.37761500	-0.93040600	1.37593000	
H	2.94839100	0.39542000	0.25145200	
H	2.22458000	0.19466800	-1.19507600	
O	-0.12328900	2.18191600	0.02031800	
H	0.76917200	1.78345500	-0.04022400	

Geometry – TS^{1BuA}2-5 – C_1				Frequency (cm^{-1})
C	-1.68045500	-0.61234100	0.26410400	-1165.51
C	-0.58269900	-1.20015500	-0.59960700	84.91 160.81 192.58 235.66 257.91 288.47
C	0.76435800	-1.30755000	0.11639000	354.07 399.73 515.37 662.00 672.42 795.54
C	1.30931400	0.01607900	0.63931500	823.93 899.27 934.78 947.41 990.62 1045.64
N	1.44395700	0.98752700	-0.44894100	1059.18 1072.90 1135.24 1164.45 1205.99 1259.42
H	-1.57971900	0.57669000	0.31034000	1297.80 1309.89 1326.99 1370.38 1393.04 1426.20
H	-2.68145300	-0.75611600	-0.15008000	1432.68 1448.84 1467.36 1474.52 1512.30 1645.12
H	-1.64442700	-0.94813200	1.30664800	2998.03 3016.52 3029.23 3053.24 3057.80 3066.61
H	-0.47830600	-0.59900100	-1.51238200	3078.15 3143.24 3532.63 3594.11 3618.50
H	-0.87965600	-2.20500300	-0.93008300	
H	1.50418200	-1.75556100	-0.56275300	
H	0.66808600	-1.99701600	0.96566800	
H	2.26001300	-0.17293700	1.16053100	
H	0.61488000	0.43951100	1.37761300	
H	1.95122300	1.80898600	-0.14126200	
H	1.96095000	0.58856500	-1.22500000	
O	-1.28164000	1.87949400	0.04873600	
H	-0.41345900	1.69517000	-0.36776600	

S1.2 2BuA + H/OH

Geometry – 2BuA – C_1				Frequency (cm^{-1})
C	-0.72058000	-0.72192500	-0.23505500	120.04 225.94 229.36 265.73 320.32 374.10
H	-0.71637600	-1.74282100	0.16908900	455.05 478.61 773.01 818.14 854.38 948.83
H	-0.58923300	-0.80172500	-1.32267600	977.23 1015.05 1038.40 1077.29 1168.43 1202.67
C	-2.04955900	-0.04622100	0.08250000	1252.20 1291.85 1328.64 1377.54 1396.04 1398.87
H	-2.13869500	0.91907000	-0.42790600	1422.88 1464.74 1477.23 1485.07 1487.20 1500.19
H	-2.89705800	-0.65906900	-0.23915500	1655.16 2932.13 3025.72 3030.51 3032.11 3073.24
H	-2.15442200	0.12678100	1.16103900	3106.83 3110.55 3120.08 3122.94 3542.45 3626.99
C	0.48389100	0.02362200	0.33372000	
H	0.37701900	0.03925000	1.43597800	
C	1.77876500	-0.69485700	-0.01401900	
H	1.90001400	-0.73244700	-1.10250300	
H	2.64749800	-0.17608500	0.40772400	
H	1.77477800	-1.71828000	0.37489800	
N	0.53256500	1.37149000	-0.22915700	
H	-0.25800900	1.92683800	0.07457800	
H	1.37142100	1.85434700	0.07015700	

Geometry – TS^{2BuA}1-1-1 – C_1				Frequency (cm^{-1})
C	0.77625200	-0.64051800	-0.64051800	-1520.01
H	0.79537100	-1.72792000	-1.72792000	96.69 185.13 221.02 240.29 264.22 351.54
H	0.62631700	-0.47383900	-0.47383900	384.22 469.00 523.05 702.28 780.22 818.49
C	2.09119400	-0.01954900	-0.01954900	933.52 968.51 1004.82 1041.51 1058.15 1065.76
H	2.07531900	1.06461200	1.06461200	1148.88 1182.93 1220.99 1285.41 1296.93 1340.20
H	2.93829900	-0.43081000	-0.43081000	1342.86 1391.88 1400.11 1406.07 1469.64 1476.40
H	2.26541900	-0.21166200	-0.21166200	1480.66 1482.64 1495.13 1737.50 2951.10 3016.83
C	-0.43669900	-0.07196700	-0.07196700	3027.85 3038.53 3069.34 3111.11 3114.08 3116.74
H	-0.26795100	-0.19448100	-0.19448100	3134.58 3459.36
C	-1.71791800	-0.81171300	-0.81171300	
H	-1.89029200	-0.72492400	-0.72492400	
H	-2.58756100	-0.39266300	-0.39266300	
H	-1.64854500	-1.87501000	-1.87501000	
N	-0.49923000	1.36784300	1.36784300	
H	-1.39728700	1.71286400	1.71286400	
H	-0.67668000	1.50570600	1.50570600	
H	-1.01477700	1.43571100	1.43571100	

Geometry – TS^{2BuA}1-1-2 – C_1				Frequency (cm^{-1})
C	0.71545600	-0.72959600	0.27121000	-1505.17
H	0.71427500	-1.78892000	-0.01692800	75.74 175.66 222.15 233.36 277.99 365.09
H	0.55758900	-0.69783600	1.35877400	383.97 473.42 515.34 711.90 777.15 814.57
C	2.05612200	-0.09846200	-0.08087600	937.17 966.37 1010.86 1019.46 1050.20 1082.52
H	2.15631000	0.90504200	0.34787500	1152.36 1195.54 1221.33 1270.54 1299.66 1333.72
H	2.88788700	-0.69534600	0.30464100	1345.02 1383.65 1400.75 1415.17 1466.20 1475.61
H	2.18153500	-0.01631000	-1.16783600	1483.17 1483.81 1498.25 1755.58 2953.37 3022.89
C	-0.48733100	-0.05351800	-0.40175200	3030.95 3033.84 3071.13 3106.16 3112.43 3119.32
H	-0.37057800	-0.17963700	-1.49478100	3137.96 3472.67

C	-1.79312200	-0.69815900	0.03715000	
H	-1.92996200	-0.55478800	1.11648700	
H	-2.64309400	-0.24145200	-0.47681600	
H	-1.78979300	-1.77394100	-0.16556300	
N	-0.57913600	1.38437000	-0.19652100	
H	-0.60252800	1.53394000	1.06098600	
H	0.32636700	1.80912800	-0.41082900	
H	-0.38080600	1.48794000	1.96524500	
Geometry – TS^{2BuA}1-2 – C_1				Frequency (cm^{-1})
C	-0.73005900	-0.73057100	-0.30027800	-1301.89
H	-0.70643000	-1.73552300	0.14012600	119.28 207.05 214.17 245.48 258.54 275.03
H	-0.63422800	-0.85166100	-1.38906000	345.34 375.27 460.03 485.55 764.50 793.62
C	-2.04819800	-0.04629300	0.04358600	835.73 961.66 986.79 1016.14 1050.92 1078.95
H	-2.17100100	0.89236600	-0.50803300	1159.94 1221.86 1239.91 1276.90 1317.95 1339.66
H	-2.90298400	-0.68049400	-0.20854500	1385.11 1397.79 1412.95 1417.11 1460.58 1475.16
H	-2.10019500	0.17523300	1.11702400	1481.72 1486.66 1498.98 1653.58 3019.36 3029.72
C	0.48461100	0.02756300	0.20899500	3030.78 3069.76 3106.35 3107.31 3122.33 3123.89
H	0.36073800	-0.00220000	1.41255500	3557.78 3648.56
C	1.79167700	-0.67673300	-0.08356200	
H	1.94639900	-0.74712300	-1.16712800	
H	2.64009700	-0.13354400	0.34810300	
H	1.78683500	-1.68701500	0.33706800	
N	0.51087400	1.37675300	-0.26770800	
H	-0.31387500	1.90266900	-0.00995900	
H	1.33215600	1.87938800	0.04273100	
H	0.19818100	-0.09316400	2.54662100	
Geometry – TS^{2BuA}1-3-1 – C_1				Frequency (cm^{-1})
C	-0.88337500	-0.01199500	0.63586500	-1505.67
H	-1.27795700	1.15912300	0.94703000	103.23 183.27 244.41 269.56 280.49 289.93
H	-1.12199000	-0.58645900	1.53679900	306.10 394.75 454.29 582.29 800.21 834.41
C	-1.67764800	-0.42739700	-0.57750200	861.68 940.65 993.15 1006.16 1033.93 1093.67
H	-1.52161100	-1.49040100	-0.80306500	1129.22 1170.34 1236.50 1247.35 1267.46 1352.50
H	-2.75062900	-0.28289800	-0.41655600	1370.93 1380.58 1388.50 1400.75 1417.72 1469.44
H	-1.36985800	0.15214100	-1.45287400	1475.89 1482.76 1493.69 1649.46 2953.87 3029.29
C	0.61401600	0.15503100	0.44567400	3035.37 3090.30 3100.36 3114.21 3129.76 3135.04
H	1.04127900	0.43865300	1.42425200	3535.32 3625.13
C	1.26678500	-1.15142200	0.00214100	
H	0.88891600	-1.44309100	-0.98339600	
H	2.35425300	-1.03485900	-0.07203500	
H	1.05959600	-1.95643800	0.71469900	
N	0.85129900	1.17071500	-0.57681300	
H	0.49730100	2.07231900	-0.27641700	
H	1.84126200	1.27351800	-0.76378800	
H	-1.51832600	2.11808200	1.14596600	
Geometry – TS^{2BuA}1-3-2 – C_1				Frequency (cm^{-1})
C	0.76118000	-0.49348100	-0.33826100	-1539.65

H	0.64239000	-0.59762600	-1.42210300	94.61 187.38 222.86 260.30 296.11 303.26
H	0.85405600	-1.70208100	0.05021100	314.30 390.06 443.52 498.85 817.79 836.70
C	2.03628000	0.18719700	0.08809500	856.44 947.96 970.45 1022.32 1065.23 1096.24
H	2.10920300	0.23710000	1.18079000	1158.29 1170.23 1234.51 1257.03 1268.45 1310.19
H	2.91654500	-0.34422100	-0.28637200	1366.86 1388.43 1394.98 1404.66 1415.39 1474.05
H	2.08599900	1.21412000	-0.29591600	1478.15 1482.72 1489.01 1653.01 2935.50 3027.52
C	-0.51447000	-0.00525000	0.31269300	3034.67 3086.05 3098.90 3113.37 3122.54 3130.82
H	-0.36565500	-0.05043800	1.40905700	3535.11 3624.12
C	-0.81383700	1.44210600	-0.06678000	
H	-0.90618500	1.52950200	-1.15494600	
H	-1.75794000	1.77075000	0.38309600	
H	-0.02450000	2.11561000	0.28084200	
N	-1.61528200	-0.84944400	-0.13843100	
H	-1.45224800	-1.81795600	0.11436000	
H	-2.49034400	-0.56360000	0.28408300	
H	0.88073100	-2.62848400	0.45143300	
Geometry – TS^{2BuA}1-4 – C_1				Frequency (cm^{-1})
C	-0.62688400	-0.82208700	-0.22500100	-1183.51
H	-0.64073600	-1.77089600	0.32549500	92.73 162.18 230.93 250.32 303.34 331.42
H	-0.40180000	-1.05855800	-1.27720600	383.80 461.81 485.75 548.82 782.34 836.88
C	-1.97154000	-0.14796600	-0.14117200	867.86 954.35 988.89 1011.95 1040.54 1080.92
H	-2.03738000	0.39375700	1.06209200	1148.11 1193.18 1214.94 1239.80 1258.92 1264.57
H	-2.09440000	0.71681100	-0.79610100	1329.22 1380.11 1392.43 1418.98 1445.75 1459.58
H	-2.84148300	-0.80470700	-0.16670000	1476.28 1486.79 1549.34 1655.58 2950.38 3003.06
C	0.51302900	0.04344400	0.31797400	3031.75 3074.03 3096.16 3110.99 3122.34 3178.60
H	0.36607600	0.13342100	1.41047000	3534.97 3617.66
C	1.85760900	-0.61678700	0.05996600	
H	2.02798600	-0.70270700	-1.01930500	
H	2.67723100	-0.02991000	0.49012100	
H	1.89223900	-1.61831400	0.50079200	
N	0.48713900	1.34666100	-0.34137100	
H	-0.31008500	1.89274100	-0.03418700	
H	1.31744200	1.87841600	-0.10757200	
H	-1.99834500	0.80368800	1.93108800	
Geometry – TS^{2BuA}1-5 – C_1				Frequency (cm^{-1})
C	-0.84922000	-0.71496000	-0.22336200	-1099.64
H	-0.86963200	-1.72983700	0.19370100	116.37 147.23 215.46 243.00 299.09 315.69
H	-0.77368300	-0.81010000	-1.31426700	398.43 458.37 466.31 562.94 773.35 839.89
C	-2.12821100	0.02608000	0.15170800	858.22 948.60 993.02 1016.83 1044.40 1074.31
H	-2.19979200	0.98797200	-0.36735100	1158.59 1169.05 1213.64 1224.84 1255.78 1290.44
H	-3.01626200	-0.55263300	-0.11967200	1319.92 1382.88 1388.80 1401.76 1441.34 1465.09
H	-2.17054200	0.21532800	1.23151300	1484.20 1499.06 1594.32 1656.05 2931.04 3030.55
C	0.41371200	-0.01089500	0.28340500	3033.88 3079.97 3092.79 3110.45 3121.80 3187.01
H	0.35584500	0.01806500	1.38923200	3538.62 3624.51
C	1.64604200	-0.78200800	-0.10460600	
H	1.86882000	-0.75682700	-1.17336900	

H	2.65218400	-0.10949400	0.43409600	
H	1.73811800	-1.77462200	0.33836200	
N	0.50195200	1.32379600	-0.30096700	
H	-0.25736000	1.91397800	0.01581900	
H	1.36841100	1.77299200	-0.02585000	
H	3.29628800	0.44930300	0.86167800	
Geometry – RC^{2BuA}2 – C_1				Frequency (cm^{-1})
C	-0.95543600	-0.11999900	-0.71095100	41.60 88.39 136.58 209.33 228.29 252.05
H	-1.33187800	-0.78925900	-1.49502100	277.44 348.48 376.92 465.31 488.73 599.77
H	-0.34447900	0.64299000	-1.21551900	724.52 778.89 821.58 919.19 956.39 978.68
C	-2.12218400	0.53983100	0.01468200	1015.83 1038.39 1076.78 1172.19 1202.57 1252.63
H	-1.77753500	1.32720300	0.69441600	1291.11 1328.45 1379.07 1399.78 1403.29 1422.65
H	-2.81406800	1.00913600	-0.69053500	1471.03 1480.21 1485.52 1489.02 1500.24 1646.45
H	-2.68999900	-0.19533300	0.59914000	2960.69 3021.45 3028.37 3028.68 3071.68 3104.67
C	-0.05375900	-0.93697600	0.21185400	3107.08 3120.07 3123.48 3471.17 3533.31 3614.20
H	-0.65421000	-1.76592500	0.62778000	
C	1.12726200	-1.51475200	-0.55263600	
H	1.73304700	-0.70162100	-0.97028100	
H	1.76650600	-2.12271600	0.09795400	
H	0.78197400	-2.14934800	-1.37473900	
N	0.46143500	-0.07259100	1.28429000	
H	-0.28676500	0.23191800	1.89720800	
H	1.12711800	-0.57958800	1.85751500	
O	1.50603900	2.04880400	-0.38795400	
H	1.23663500	1.40162400	0.30799000	
Geometry – TS^{2BuA}2-1-1 – C_1				Frequency (cm^{-1})
C	1.10061800	0.15649700	-0.63476200	-612.79
H	1.51610200	0.98182700	-1.22632900	57.90 125.19 149.61 211.63 218.77 244.23
H	0.52425300	-0.47258100	-1.32731900	266.39 327.98 371.35 456.54 491.07 650.78
C	2.22324600	-0.64478000	0.01266000	710.45 783.83 819.15 937.64 976.31 1018.09
H	1.82485000	-1.48287400	0.59261500	1047.43 1063.47 1129.12 1193.64 1229.00 1304.12
H	2.90627500	-1.04880400	-0.74052500	1312.33 1355.89 1381.37 1398.62 1402.76 1470.59
H	2.80851200	-0.01756700	0.69549900	1476.03 1482.66 1487.53 1491.84 1607.83 2514.20
C	0.12146400	0.74361900	0.37702300	2973.60 3028.02 3032.80 3038.29 3078.14 3111.59
H	0.68957700	1.34362200	1.10977600	3114.43 3122.75 3128.77 3580.32 3828.45
C	-0.90408200	1.65099700	-0.30100100	
H	-1.44511700	1.07677200	-1.06110700	
H	-1.62686900	2.04392100	0.42337900	
H	-0.41004100	2.50167700	-0.78258400	
N	-0.48910700	-0.33393000	1.13559400	
H	-1.23816200	-0.01824400	1.74271500	
H	-0.79577200	-1.16798100	0.55582500	
O	-1.74465000	-1.45831900	-0.54978300	
H	-2.62014000	-1.17369900	-0.25635800	
Geometry – TS^{2BuA}2-1-2 – C_1				Frequency (cm^{-1})
C	-1.01574700	-0.40795900	-0.61509600	-582.87

H	-1.39664000	-1.32092600	-1.09083700	56.76 123.87 161.52 205.41 229.57 249.17
H	-0.54161800	0.19794100	-1.39770100	276.74 343.96 381.00 464.85 487.09 658.71
C	-2.16447900	0.35716400	0.02816300	723.50 778.21 820.21 941.35 970.69 1005.95
H	-1.84469500	1.34471800	0.38245900	1025.74 1077.89 1142.18 1199.02 1242.46 1285.55
H	-2.97855100	0.52359300	-0.68333900	1313.46 1349.68 1380.13 1398.76 1411.04 1465.92
H	-2.57862100	-0.18958400	0.88481700	1476.60 1483.71 1490.64 1496.88 1607.41 2470.39
C	0.08050800	-0.82177600	0.37784900	2977.02 3025.29 3035.49 3039.10 3080.54 3101.50
H	-0.37675200	-1.51368100	1.10694500	3121.17 3121.73 3135.33 3581.39 3828.16
C	1.22838300	-1.51543900	-0.33755000	
H	1.69905100	-0.81015700	-1.03285200	
H	1.98361700	-1.86110600	0.37403200	
H	0.86463300	-2.37428800	-0.90982100	
N	0.61299700	0.27669600	1.16038900	
H	1.29589600	0.90588900	0.63688500	
H	-0.10425200	0.82181100	1.62721200	
O	1.29227400	1.78094800	-0.53989000	
H	0.57677700	2.41940200	-0.42160000	
Geometry – TS^{2BuA}2-2 – C_1				Frequency (cm^{-1})
C	0.46380600	-0.91332800	-0.75321500	-238.31
H	0.57588600	-0.45390600	-1.74400100	20.88 65.89 86.12 127.59 202.65 231.89
H	0.04593800	-1.91803000	-0.90726100	269.86 331.12 373.80 454.43 484.43 524.19
C	1.82237400	-0.99800400	-0.06715500	756.38 799.77 830.39 953.57 969.33 1013.68
H	1.77212400	-1.59534600	0.85005200	1039.63 1078.60 1167.50 1207.12 1231.06 1285.91
H	2.56579000	-1.47111100	-0.71555100	1312.45 1353.64 1383.86 1398.17 1407.72 1461.17
H	2.18328000	0.00518000	0.19059400	1472.66 1479.98 1487.73 1498.55 1655.28 2225.06
C	-0.55107200	-0.09131800	0.03415900	3023.17 3033.32 3034.13 3070.54 3111.78 3113.04
H	-0.10938400	0.95363000	0.11370400	3121.28 3123.01 3553.71 3641.60 3802.85
C	-1.88205400	0.01913500	-0.68983400	
H	-2.32495400	-0.97710800	-0.80307700	
H	-2.58751700	0.64580300	-0.13279500	
H	-1.74946400	0.45681800	-1.68448300	
N	-0.75211400	-0.65012600	1.34759700	
H	-1.45373300	-0.14110800	1.87022000	
H	0.10151800	-0.66606800	1.89101300	
O	0.81962200	2.36909800	0.15393700	
H	0.57001800	2.66043600	-0.73682400	
Geometry – TS^{2BuA}2-3-1 – C_1				Frequency (cm^{-1})
C	0.75201300	0.23009500	-0.60079500	-1061.31
H	0.40231300	1.21656400	-0.02831100	107.83 136.98 180.55 191.75 246.42 271.73
H	0.74375600	0.52886700	-1.65721900	315.17 390.52 447.55 486.00 572.87 636.53
C	2.14021100	-0.13813100	-0.12495800	779.34 824.35 880.00 946.77 985.48 1001.01
H	2.44666300	-1.11025500	-0.53573800	1011.84 1068.78 1111.08 1149.01 1183.86 1251.44
H	2.87654600	0.60565100	-0.44059100	1319.30 1328.23 1363.96 1378.91 1397.12 1419.27
H	2.18259700	-0.20955700	0.96633500	1469.01 1475.03 1482.88 1494.39 1535.89 1644.53
C	-0.33042200	-0.80356700	-0.33009500	2935.23 3023.63 3035.35 3056.34 3103.42 3114.36
H	-0.08136000	-1.70697000	-0.91956800	3129.71 3135.18 3533.54 3620.48 3682.72

C	-0.40135300	-1.19173100	1.14050100	
H	-0.52440700	-0.29386600	1.75684200	
H	-1.25061100	-1.85976900	1.32287200	
H	0.50567600	-1.71538200	1.45577300	
N	-1.62159300	-0.23473200	-0.72792600	
H	-1.63675900	-0.02281300	-1.71963400	
H	-2.37697600	-0.88413600	-0.54282100	
O	-0.45567400	2.11122600	0.57920200	
H	-1.25359100	1.62500000	0.29600600	
Geometry – TS^{2BuA}2-3-2 – C_1				Frequency (cm^{-1})
C	-0.65134700	-0.29889100	-0.71064200	-1121.73
H	-0.60029000	-0.51514800	-1.78435500	114.65 126.38 183.30 201.64 236.29 269.19
H	-0.68765300	0.89351700	-0.63850800	301.93 370.79 453.45 479.13 573.68 688.99
C	-1.91720200	-0.82632400	-0.07057000	808.92 879.04 904.35 953.08 991.02 1009.66
H	-2.04943600	-0.41945700	0.93820400	1032.78 1043.67 1085.58 1182.91 1199.75 1229.00
H	-2.80220700	-0.54764400	-0.64871600	1256.36 1318.42 1361.02 1388.55 1399.50 1415.42
H	-1.89234000	-1.92263600	0.00071400	1469.35 1479.52 1486.72 1487.45 1562.30 1646.94
C	0.63911400	-0.70700200	-0.01857300	2936.30 3021.76 3035.22 3074.67 3099.67 3115.72
H	0.70352400	-1.81147700	-0.06031600	3130.19 3131.21 3540.48 3623.27 3674.89
C	1.85027100	-0.11824500	-0.72639100	
H	1.78547300	0.97543500	-0.72226100	
H	2.78152800	-0.41712500	-0.23195100	
H	1.89475200	-0.46033000	-1.76498700	
N	0.61976100	-0.22345600	1.36611100	
H	-0.06749600	-0.71844300	1.92251100	
H	1.52153600	-0.36404300	1.80618800	
O	-0.54304400	2.10715600	-0.00042800	
H	-0.10637700	1.71706700	0.78117900	
Geometry – TS^{2BuA}2-4 – C_1				Frequency (cm^{-1})
C	0.14789200	1.14647000	-0.73211300	-1171.82
H	-0.09975800	0.96870000	-1.78742500	69.99 154.52 219.13 269.81 291.22 306.21
H	0.66968900	2.11438000	-0.70153000	357.51 378.38 415.51 565.28 665.65 714.76
C	-1.12204200	1.26188900	0.08629000	803.90 831.75 906.78 946.47 962.71 1011.34
H	-1.67675900	0.20149700	0.20277800	1031.07 1057.33 1082.33 1165.91 1179.69 1252.88
H	-0.94721200	1.58151800	1.11727400	1273.86 1306.31 1371.57 1382.06 1386.96 1422.13
H	-1.87464100	1.90008800	-0.38347700	1436.48 1453.03 1463.25 1480.02 1497.49 1642.65
C	1.13491900	0.06345900	-0.27375400	2975.02 3016.60 3034.55 3060.00 3081.81 3114.86
H	2.11252000	0.30207200	-0.72517200	3124.07 3153.59 3528.91 3598.55 3614.42
C	1.29917100	0.02983300	1.23883500	
H	0.36583400	-0.29312400	1.71393100	
H	2.09008600	-0.67039400	1.52985800	
H	1.56622400	1.01923900	1.62526200	
N	0.66033600	-1.25330900	-0.72182100	
H	0.64457200	-1.30446900	-1.73464100	
H	1.28057200	-1.98548200	-0.39330400	
O	-2.04014700	-1.08149600	0.33662000	

H	-1.19195200	-1.41879800	-0.01932300	
Geometry – TS^{2BuA}2-5 – C_1				Frequency (cm^{-1})
C	-1.00185400	0.42013000	-0.64454200	-1293.52
H	-1.15554600	0.31826100	-1.72636900	87.98 113.82 191.43 219.77 262.31 304.36
H	-0.43516700	1.34703400	-0.48684100	360.96 408.57 448.69 475.78 559.57 713.27
C	-2.35039900	0.49074200	0.06253100	776.39 829.44 900.60 937.78 964.48 988.99
H	-2.23975800	0.74679300	1.12219400	1030.11 1068.47 1078.26 1174.62 1189.22 1249.28
H	-2.99047400	1.25770600	-0.38319100	1286.48 1296.64 1322.52 1369.70 1400.79 1418.04
H	-2.88304700	-0.46633000	-0.00387300	1445.61 1471.75 1484.13 1498.65 1511.59 1647.85
C	-0.13840400	-0.75160600	-0.18150900	2926.29 3028.59 3037.89 3076.73 3081.75 3106.91
H	-0.67888800	-1.68767500	-0.42532700	3123.07 3156.57 3538.53 3623.90 3693.80
C	1.18819600	-0.76971900	-0.90742000	
H	1.09254600	-0.69625200	-1.99372400	
H	1.78864000	0.22494800	-0.55843400	
H	1.83891000	-1.60196600	-0.62304200	
N	0.11780400	-0.63866200	1.25739400	
H	-0.74606600	-0.65832600	1.78582500	
H	0.68656200	-1.41274300	1.58270200	
O	2.14447300	1.22572400	0.24479500	
H	1.55664300	0.95610400	0.97560800	

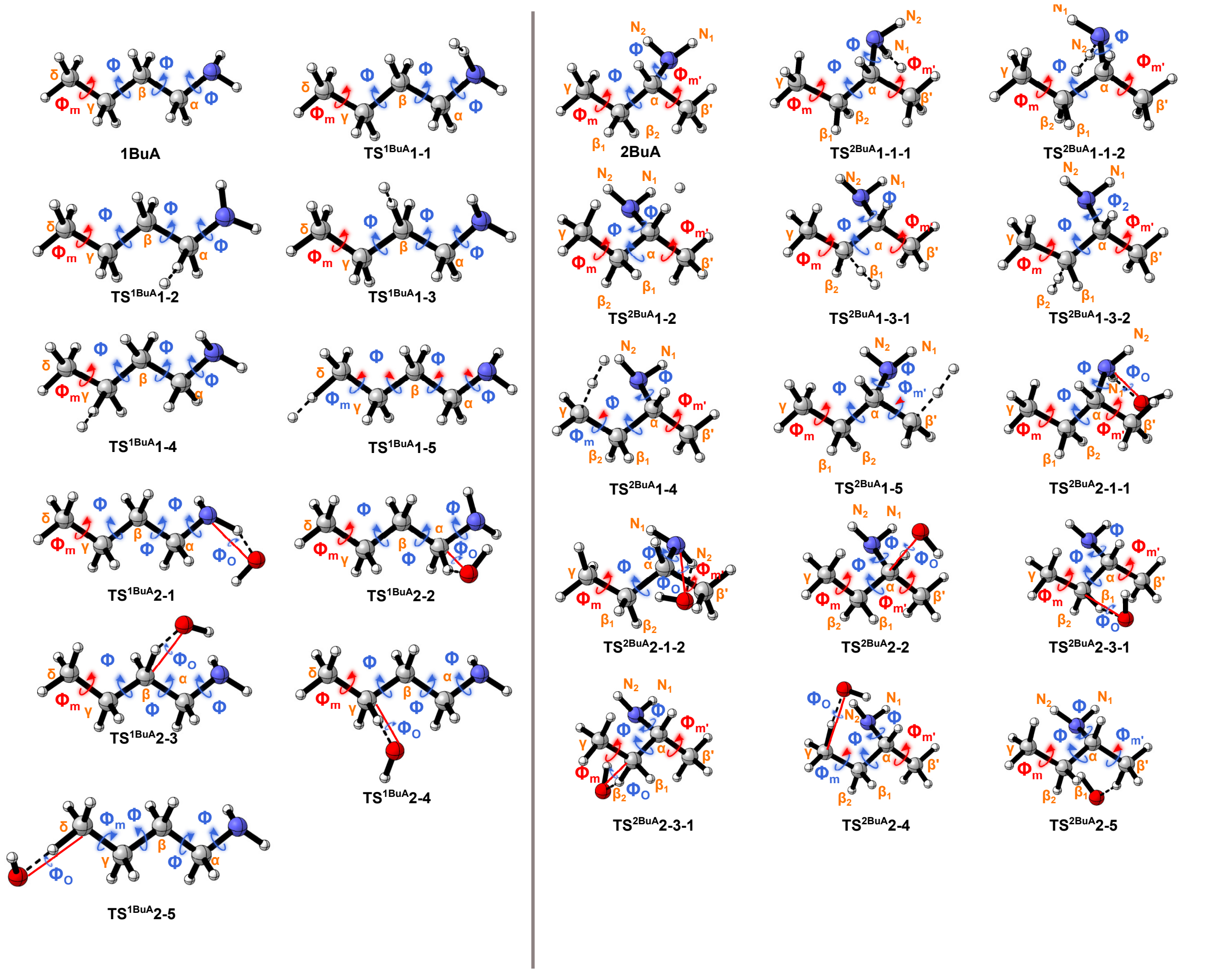

Fig. S1.: Representation of dihedral angles used in the conformational search. Carbon, nitrogen, and oxygen are represented by gray, blue, and red, respectively. Dihedrals of methyl groups, represented by red arrows, are not included in the conformational search.

S2. Rate Constants - 1BuA + H/OH

	R1-1	R1-2	R1-3	R1-4	R1-5
250	1.08E+06	1.47E+10	3.98E+07	2.27E+07	1.04E+06
298	7.64E+06	3.24E+10	2.43E+08	1.29E+08	1.09E+07
350	4.37E+07	6.53E+10	1.12E+09	5.59E+08	7.91E+07
400	1.72E+08	1.12E+11	3.63E+09	1.71E+09	3.59E+08
450	5.39E+08	1.83E+11	9.49E+09	4.28E+09	1.23E+09
500	1.41E+09	2.53E+11	2.12E+10	9.21E+09	3.44E+09
600	6.44E+09	5.22E+11	7.66E+10	3.12E+10	1.72E+10
700	2.04E+10	9.39E+11	2.05E+11	7.93E+10	5.82E+10
800	5.08E+10	1.57E+12	4.48E+11	1.67E+11	1.52E+11
900	1.06E+11	2.44E+12	8.53E+11	3.10E+11	3.31E+11

1000	1.97E+11	3.61E+12	1.47E+12	5.22E+11	6.34E+11
1200	5.24E+11	6.93E+12	3.47E+12	1.21E+12	1.77E+12
1400	1.11E+12	1.18E+13	6.74E+12	2.31E+12	3.89E+12
1600	2.00E+12	1.82E+13	1.15E+13	3.95E+12	7.26E+12
1800	3.26E+12	2.65E+13	1.79E+13	6.17E+12	1.21E+13
2000	4.92E+12	3.65E+13	2.60E+13	9.05E+12	1.86E+13

	R2-1	R2-2	R2-3	R2-4	R2-5
250	1.98E+12	1.06E+12	9.02E+11	2.81E+11	4.77E+10
298	1.73E+12	9.55E+11	4.29E+11	1.48E+11	2.67E+10
350	1.66E+12	9.26E+11	2.95E+11	1.06E+11	2.63E+10
400	1.68E+12	9.41E+11	2.60E+11	9.80E+10	3.47E+10
450	1.75E+12	9.86E+11	2.60E+11	1.04E+11	5.01E+10
500	1.86E+12	1.05E+12	2.79E+11	1.20E+11	7.25E+10
600	2.15E+12	1.24E+12	3.51E+11	1.75E+11	1.42E+11
700	2.51E+12	1.51E+12	4.61E+11	2.58E+11	2.50E+11
800	2.93E+12	1.84E+12	6.08E+11	3.70E+11	4.04E+11
900	3.42E+12	2.25E+12	7.91E+11	5.12E+11	6.11E+11
1000	3.97E+12	2.74E+12	1.01E+12	6.87E+11	8.76E+11
1200	5.26E+12	3.96E+12	1.58E+12	1.14E+12	1.61E+12
1400	6.79E+12	5.56E+12	2.33E+12	1.73E+12	2.63E+12
1600	8.58E+12	7.56E+12	3.27E+12	2.48E+12	3.98E+12
1800	1.06E+13	9.98E+12	4.41E+12	3.39E+12	5.67E+12
2000	1.29E+13	1.29E+13	5.75E+12	4.46E+12	7.73E+12

S3. Rate Constants - 2BuA + H/OH

	R1-1-1	R1-1-2	R1-2	R1-3-1	R1-3-2	R1-4	R1-5
250	3.26E+05	3.12E+05	5.99E+10	2.62E+07	3.73E+07	1.68E+06	5.26E+05
298	2.76E+06	2.76E+06	1.17E+11	1.78E+08	2.25E+08	1.83E+07	6.63E+06
350	1.81E+07	1.87E+07	2.13E+11	8.78E+08	1.02E+09	1.36E+08	5.58E+07
400	7.89E+07	8.31E+07	3.48E+11	2.94E+09	3.26E+09	6.27E+08	2.81E+08
450	2.67E+08	2.85E+08	5.32E+11	7.87E+09	8.44E+09	2.17E+09	1.05E+09
500	7.46E+08	8.02E+08	7.74E+11	1.79E+10	1.87E+10	6.09E+09	3.12E+09
600	3.81E+09	4.13E+09	1.46E+12	6.55E+10	6.64E+10	3.07E+10	1.73E+10

700	1.32E+10	1.43E+10	2.46E+12	1.76E+11	1.76E+11	1.04E+11	6.31E+10
800	3.54E+10	3.83E+10	3.81E+12	3.87E+11	3.82E+11	2.73E+11	1.75E+11
900	7.93E+10	8.54E+10	5.57E+12	7.39E+11	7.24E+11	5.97E+11	3.99E+11
1000	1.56E+11	1.67E+11	7.83E+12	1.27E+12	1.24E+12	1.15E+12	7.94E+11
1200	4.55E+11	4.86E+11	1.39E+13	3.02E+12	2.95E+12	3.23E+12	2.36E+12
1400	1.04E+12	1.10E+12	2.21E+13	5.89E+12	5.76E+12	7.14E+12	5.41E+12
1600	2.00E+12	2.13E+12	3.26E+13	1.01E+13	9.88E+12	1.34E+13	1.05E+13
1800	3.44E+12	3.65E+12	4.55E+13	1.57E+13	1.54E+13	2.25E+13	1.80E+13
2000	5.43E+12	5.74E+12	6.07E+13	2.28E+13	2.25E+13	3.49E+13	2.83E+13

	R1-1-1	R1-1-2	R1-2	R1-3-1	R1-3-2	R1-4	R1-5
250	1.08E+12	1.00E+12	1.46E+12	2.28E+11	8.91E+11	6.87E+10	1.83E+11
298	9.34E+11	8.53E+11	1.12E+12	1.35E+11	3.75E+11	4.84E+10	1.21E+11
350	8.77E+11	7.96E+11	9.50E+11	1.10E+11	2.49E+11	4.46E+10	1.11E+11
400	8.26E+11	7.90E+11	8.75E+11	1.10E+11	2.22E+11	4.94E+10	1.21E+11
450	8.15E+11	8.09E+11	8.46E+11	1.22E+11	2.28E+11	6.04E+10	1.43E+11
500	8.28E+11	8.50E+11	8.46E+11	1.42E+11	2.51E+11	7.75E+10	1.76E+11
600	8.85E+11	9.71E+11	9.00E+11	2.01E+11	3.33E+11	1.32E+11	2.72E+11
700	9.83E+11	1.13E+12	1.00E+12	2.83E+11	4.52E+11	2.19E+11	4.12E+11
800	1.15E+12	1.33E+12	1.15E+12	3.89E+11	6.08E+11	3.44E+11	6.05E+11
900	1.34E+12	1.60E+12	1.33E+12	5.21E+11	8.01E+11	5.14E+11	8.56E+11
1000	1.57E+12	1.91E+12	1.54E+12	6.79E+11	1.03E+12	7.36E+11	1.17E+12
1200	2.19E+12	2.69E+12	2.07E+12	1.08E+12	1.63E+12	1.36E+12	2.03E+12
1400	2.99E+12	3.73E+12	2.75E+12	1.61E+12	2.40E+12	2.26E+12	3.22E+12
1600	4.01E+12	4.47E+12	3.58E+12	2.26E+12	3.37E+12	3.48E+12	4.77E+12
1800	5.27E+12	5.88E+12	4.56E+12	3.05E+12	4.53E+12	5.06E+12	6.73E+12
2000	6.78E+12	7.55E+12	5.71E+12	3.98E+12	5.91E+12	7.02E+12	9.11E+12

S4. Fit of rate constants

The four-parameter equation used in this study, proposed by Zheng and Truhlar, is given below:

$$k = A\left(\frac{T}{300}\right)^n \exp\left[-B\frac{T+T_0}{T^2+T_0^2}\right] \tag{1}$$

where A, n, B, and T_0 are the fitting parameters. Table S1. lis the fitting parameter for 1- and 2-butylamine + H/OH reactions.

Table S1.: Fitting Parameters to the PEM//MS-CVT/SCT rate constant at 250-2000 K.

	A (molecule cm^{-3} s^{-1})	B (K)	n	T_0 (K)
R^{1BuA}1-1	3.404(-13)	2581	2.417	179.4
R^{1BuA}1-2	1.901(-13)	316.8	3.152	176.5
R^{1BuA}1-3	9.954(-13)	1940	2.547	149.8
R^{1BuA}1-4	2.295(-13)	1722	2.693	135.2
R^{1BuA}1-5	8.525(-13)	2668	2.656	145.7
R^{1BuA}2-1	4.081(-10)	2849	-0.700	707.6
R^{1BuA}2-2	2.174(-10)	3325	-0.198	822.7
R^{1BuA}2-3	2.054(-06)	7482	-0.425	614.1
R^{1BuA}2-4	6.146(-07)	7192	-0.383	591.2
R^{1BuA}2-5	2.385(-06)	7507	-0.424	507.9
R^{2BuA}1-1-1	1.639(-13)	2640	2.874	173.0
R^{2BuA}1-1-2	1.851(-13)	2658	2.841	167.0
R^{2BuA}1-2	8.449(-13)	383.1	2.640	213.1
R^{2BuA}1-3-1	8.738(-13)	1974	2.552	134.6
R^{2BuA}1-3-2	6.768(-13)	1859	2.650	144.8
R^{2BuA}1-4	1.410(-12)	2659	2.716	141.2
R^{2BuA}1-5	1.107(-12)	2852	2.791	142.3
R^{2BuA}2-1-1	1.694(-10)	3967	-0.192	1010
R^{2BuA}2-1-2	1.241(-10)	3131	-0.244	846.6
R^{2BuA}2-2	3.538(-10)	3838	-0.717	881.1
R^{2BuA}2-3-1	3.004(-09)	4908	-1.750	626.5
R^{2BuA}2-3-2	1.498(-07)	6475	-3.129	634.4
R^{2BuA}2-4	2.094(-09)	5041	-1.219	598.1
R^{2BuA}2-5	1.791(-09)	4643	-1.118	619.9

S5. CCUS Rate Constant

The individual competitive CUS, that is, for each reaction, was calculated using the equation below:[1]

$$k_i^{\text{CCUS}} = \frac{k_a \sum k_i}{k_a + \sum k_i} \alpha_i \tag{2}$$

where $\sum k_i$ is the sum of the rate constants that proceed through each transition state, calculated using the PEM//MS-CVT/SCT approach, and α_i is the ratio between the individual rate constant for a given transition state and the total sum of all such constants. Here, k_a represents the association rate constant, which was calculated using the expression proposed by Klippenstein:[2]

$$k_a = k^{\text{LR-TST}} = C\mu^{-1/2}(d_{\text{OH}}d_{\text{1BuA/2BuA}})^{2/3}T^{-1/6} \quad (3)$$

where C = 1.8 × 10^{-09}, μ is the reduced mass, and d_{OH} = 1.812, d_{1BuA} = 1.2868, and d_{2BuA} = 1.2208.

[1] González, D.; Canosa, A..; Martínez-Núñez, E.; Fernández-Ramos, A.; Ballesteros, B.; Agúndez, M.; Cernicharo, J.; Jiménez, E. Effect of temperature on the gas-phase reaction of CH3CN with OH radicals: experimental (T= 11.7–177.5 K) and computational (T= 10–400 K) kinetic study. Phys. Chem. Chem. Phys., 2024,26, 3632-3646

[2] Georgievskii, Y.; Klippenstein, S. J. Long-range transition state theory. J. Chem. Phys., 2005, 122, 194103;
